# Patient Stratification for Improving Acute Chest Pain Management and Mitigating Emergency Department Crowding: Machine Learning Model Development and Validation

**DOI:** 10.2196/83099

**Published:** 2026-08-21

**Authors:** Chih-Chia Hsieh, Shao-Chung Chu, Jung-Ting Lee, Chih-Hao Lin, Chung-Yao Kao

**Affiliations:** 1 Department of Emergency Medicine, National Cheng Kung University Hospital, Tainan, Taiwan; 2 School of Medicine, National Sun Yat-Sen University, No.70 Lien-hai Road, Kaohsiung, 804, Taiwan, 886 75252000; 3 Department of Electrical Engineering, National Sun Yat-Sen University, Kaohsiung, Taiwan

**Keywords:** acute chest pain, risk stratification, emergency department crowding, clinical decision support, triage, patient management, AI

## Abstract

**Background:**

Acute chest pain (ACP) is one of the most common chief complaints in the emergency department (ED), accounting for approximately 8% of all ED visits. However, among patients presenting with chest pain suggestive of cardiac origin, fewer than 10% are ultimately diagnosed with acute coronary syndrome (ACS).

**Objective:**

AI models were developed to support clinical decision-making for triage level-2 ED patients presenting with ACP. These models aim to accurately detect ACS and reliably identify low-risk patients based on a single high-sensitivity cardiac troponin T test result. By integrating this AI-assisted strategy into clinical workflows, we aim to reduce ED length of stay, alleviate crowding, and improve health care efficiency while maintaining high safety standards.

**Methods:**

We conducted a retrospective study using single-center data from a tertiary teaching hospital between January 2016 and December 2022. Models based on artificial neural networks (ANNs) were trained to classify patients with ACP at triage level 2 into 3 clinical classes: critical patients with ACS (subgroup GA), critical patients without ACS (subgroup GB1), and low-risk patients (subgroup GB2). The models were trained and internally validated via a 5-fold cross-validation protocol using data from 2016 to 2020. Model performance was then evaluated on the hold-out testing data (2021‐2022) using area under the receiver operating characteristic curve, area under the precision-recall curve, and subgroup-specific metrics. Feature selection methods and the Shapley Additive Explanations value analysis were applied to identify features that drive the model’s predictive power. The study was approved by the institutional review board of the National Cheng Kung University Hospital, Tainan, Taiwan (A-ER-111‐199).

**Results:**

After excluding ED visits with missing triage data, incomplete medical histories, or unsuitable dispositions, 17,935 visits were included, with 1209 GA, 3873 GB1, and 12,853 GB2. Rendering prediction based on 24 feature variables (2 demographics, 6 vital signs, 4 blood test results, and 12 medical history), all ANN models demonstrated strong testing AUROC (95% CI) performance of 0.942 (0.920‐0.965) for GA classification, 0.824 (0.808‐0.841) for GB1, and 0.893 (0.884‐0.902) for GB2. Regarding multiclassification, ANN-S3 achieved balanced performance, with ACS sensitivity of 0.941 (95% CI 0.908‐0.973) and low-risk positive predictive value and sensitivity of 0.911 (95% CI 0.9‐0.921) and 0.837 (95% CI 0.824‐0.85), respectively. A sensitivity-prioritized variant, ANN-S3-L, increased ACS sensitivity to 0.966 (95% CI 0.94‐0.991) and negative predictive value to 0.998 (95% CI 0.996‐0.999), but at the cost of lower specificity and reduced low-risk sensitivity, indicating a safety-efficiency trade-off.

**Conclusions:**

These findings suggest that ANN-based classifiers can effectively support clinical risk stratification and disposition decision-making in ACP care for patients presenting ≥3 hours after symptom onset. However, because even the sensitivity-prioritized ANN-S3-L variant falls short of the stringent sensitivity threshold (>0.99) typically required for a standalone ED rule-out tool, this system should be interpreted as a clinical decision-support aid rather than an independent rule-out strategy.

## Introduction

Acute chest pain (ACP) is one of the most common chief complaints in the emergency department (ED), accounting for approximately 8% of all ED visits. However, among patients presenting with chest pain suggestive of cardiac origin, fewer than 10% are ultimately diagnosed with acute coronary syndrome (ACS) [1]. Despite this low diagnostic yield, current clinical practice often necessitates multiple measurements of high-sensitivity cardiac troponin T (hs-TnT) and prolonged observation in the ED to safely rule out ACS before discharge decisions can be made [2]. Although this approach enhances diagnostic sensitivity, it also prolongs the patient’s length of stay (LoS) in the ED. This extended stay is recognized as one of the major contributing factors to ED crowding [3]. ED crowding is widely acknowledged to negatively impact patient safety and overall quality of care [4]. It also imposes significant burdens on health care providers, leading to increased workload and elevated stress levels [5]. In general, ED crowding not only reduces health care system efficiency but also poses substantial risks to patient safety [6].

To reduce the LoS for patients with ACP, previous studies have proposed the single test rule-out strategy, which aims to expedite ACS exclusion by combining a single hs-TnT measurement with clinical assessment [7]. However, the successful implementation of this strategy remains limited in practice [8]. One of the primary challenges is that clinicians must rely heavily on clinical experience and judgment to accurately identify low-risk patients, in order to avoid premature discharge and associated adverse outcomes [9].

The objective of this study is to develop an AI model, whose predictive power is medically interpretable, to support clinical decision-making in the initial management of patients presenting to the ED with ACP. This is in line with several recent studies that applied explainable AI techniques to enhance interpretability and clinician trust in emergency care-focused models [10,11]. This model is designed not only to accurately detect ACS but also to rapidly and reliably identify low-risk patients based on a single hs-TnT test. By integrating this AI-assisted strategy into clinical workflows, we aim to reduce ED LoS, alleviate crowding, and ultimately improve health care efficiency while lowering medical expenditures.

## Methods

### Study Design and Setting

This study retrospectively collects data from the ED of a tertiary teaching hospital located in Tainan City, Taiwan, between January 1, 2016, and December 31, 2022. The hospital comprises 783 general ward beds and 136 intensive care unit beds, and on average, receives approximately 100,000 ED visits annually. Adult patients presenting with chest pain and triaged as level 2 (urgent) were included [12]. Inclusion required complete triage data, medical history, examination results, and documented disposition. Patients were categorized into 3 groups: group GA (ACS), group GB1 (non-ACS but requiring acute care or hospitalization), and group GB2 (noncritical, discharged after short observation). Visits with missing or indeterminate data were all excluded, which was the only exclusion criterion. As such, all included visits have no missing data. The dataset was divided chronologically: records from January 1, 2016, to December 31, 2020, were used for training and from January 1, 2021, to December 31, 2022, for testing (75/25 split). Repeat visits were all included unless with missing or indeterminate data.

To maintain the clinical integrity and analytical precision required for this pilot research, we prioritized our institutional dataset over open ED datasets, such as Medical Information Mart for Intensive Care IV Emergency Department and Multimodal Clinical Monitoring in the Emergency Department.

### Data Collection and Processing

In total, 27 variables were used for data analysis and model development. These included 3 response variables, 2 demographic variables (age and sex), and 22 clinical variables. The clinical variables consisted of 6 vital signs, 4 blood test results, and history of 12 diseases and medical conditions. Among the 27 variables, the response, sex, and medical history were categorical, while the remaining variables were quantitative (Textbox 1).

Quantitative variables were used in their raw form without prior scaling, standardization, or log-transformation. Instead, potential disparities in numerical scales were (partially) mitigated via batch normalization integrated directly within the artificial neural network (ANN) architectures. Because batch normalization primarily addresses internal covariate shift rather than correcting highly skewed raw input distributions (such as hs-TnT), a sensitivity analysis was conducted by using instead the “log-transformed hs-TnT” as an alternative input.

In alignment with Good Machine Learning Practice principles [13] regarding the clinical workflow context, we defined a precise index time anchor to prevent data leakage. The model’s prediction shall be executed at the exact moment the first laboratory panel (including hs-TnT, creatinine, and complete blood count) is reported, which typically occurs within 60 minutes of patient arrival. Only features available at or before this timestamp were used as model inputs. By anchoring the prediction at this specific decision window—after initial laboratory results are available but before final disposition or serial testing is initiated—we ensure the model functions as a true clinical decision support (CDS) tool within the intended use environment.

Textbox 1. **Definitions of variables.**

- Response variables: Each visit was linked to 3 mutually exclusive binary variables indicating group assignment (GA, GB1, or GB2), with only one variable coded as 1 per patient.
- Vital signs: Six vital signs were measured and recorded by triage nurses, including temperature, heart rate, respiratory rate, systolic and diastolic blood pressure, and peripheral oxygen saturation.
- Blood test: Four blood tests—creatinine, platelet count, white blood cell count, and high-sensitivity cardiac troponin T (measured by Elecsys assay; Roche Diagnostics)—were used for prediction. Serum high-sensitivity cardiac troponin T concentrations were measured using the Elecsys Troponin T hs STAT immunoassay on the cobas e analyzer (Roche Diagnostics). All assay results were reported in nanograms per liter. The assay is characterized by a limit of detection of 5 ng/L and an upper reference limit (99th percentile) of 14 (95% CI 12.7-24.9) ng/L. The clinical sampling protocol used in this study supports both immediate sampling (0 hours) for rapid rule-out and serial sampling at appropriate time intervals (eg, 0-3 hours or longer) to detect the characteristic rise and/or fall of troponin levels required for clinical adjudication. This approach and the associated diagnostic algorithms mirror several established international clinical guidelines, including the 2011 and 2015 European Society of Cardiology 0 hours and 1 hour and 0 hours and 3 hours algorithms, the 2014 National Institute for Health and Care Excellence guidelines for the early rule-out of non–ST-segment elevation myocardial infarction, the Third Universal Definition of Myocardial Infarction, the World Health Organization (WHO) criteria for the definition of acute myocardial infarction, and the Joint American College of Cardiology Foundation/American Heart Association/World Heart Federation task force recommendations for clinical diagnosis.
- Medical history: Twelve conditions were included as binary variables: coronary artery disease, connective tissue disease, peripheral vascular disease, peptic ulcer disease, liver disease, chronic obstructive pulmonary disease, diabetes mellitus (DM), malignancy, AIDS, congestive heart failure, cerebrovascular accident or transient ischemic attack, and dementia. All of the medical conditions considered refer strictly to the patient’s preexisting medical history (comorbidity) recorded prior to the corresponding index ED visit.

### Definition of Groups GA, GB1, and GB2

Patients diagnosed with ACS were classified as GA. Those without ACS were further categorized as GB1 if they had other serious conditions requiring hospitalization, transfer, or died in the ED, and as GB2 if they were discharged after ED care.

To ensure the safety and validity of the ground truth labels, we established a rigorous adjudication process for ACS, specifically focusing on type 1 myocardial infarction (MI) in accordance with the Fourth Universal Definition of MI [14]. To maintain high specificity and provide a fit-for-purpose reference standard, cases of unstable angina and type 2 MI were intentionally excluded from the target outcome group GA.

The diagnostic adjudication was a multidisciplinary effort. Initial clinical assessments were performed by board-certified emergency physicians, with definitive diagnosis and type 1 versus type 2 MI distinction determined by consulting cardiologists based on clinical presentation, serial electrocardiogram (ECG) changes, and biomarker trajectories. To further minimize label noise and information bias, we implemented a dual-referencing verification strategy: a visit was only labeled as GA if the corresponding *International Classification of Diseases, 10th Revision* (ICD-10 codes; I21.0-I21.4, I22.x) were documented in both the initial ED record and the final hospital discharge summary [15]. This methodology ensures that the model learns from confirmed ischemic events while excluding initial ED misdiagnoses or secondary events occurring after the index visit.

### Model Development, Fitting, and Evaluation

ANNs were developed in Python to predict patients’ categorical outcomes (GA, GB1, or GB2) [16]. Three submodels—ANN-GA, ANN-GB1, and ANN-GB2—were trained to estimate the probability of each respective group. Their outputs were integrated via a multiclassifier for final prediction. All submodels shared the same architecture, with training parameters tailored to their respective targets; therefore, only the development of ANN-GA is detailed below. The first, second, and third response variables were used to train ANN-GA, ANN-GB1, and ANN-GB2, respectively. To address the imbalance among GA, GB1, and GB2 cohorts, the approach of assigning different weights to different patient groups, or down-sampling the dominating cohort, was adopted when training our models. We did not apply the synthetic minority oversampling technique. We used the scikit-learn (0.23.2) and the tensorflow (2.8.0) libraries for training our models.

The ANN-GA model was developed following a procedure similar to our previous work [17]. A 5-fold cross-validation protocol [18] was applied to the training dataset, dividing it into 5 equal subsets. In the *i*th iteration (*i*=1, 2, ... , 5), the *i*th subset was held out for internal validation, while a model *M_i_*(*x*) of the form:


(1) $$M_{i}\left(x\right)=\frac{1}{1+e^{f_{i}\left(x\right)}}$$

(where *x* represents the input vector of 24 decision variables) was trained on the remaining 4 subsets. Central to the model *M_i_*(*x*) is the nonlinear function *f_i_*(*x*), which was obtained by training a fully connected ANN consisting of 4 layers. Each hidden layer (except the last) comprises batch normalization [19], affine transformation, and nonlinear activation. The batch normalization recenters and scales the input to stabilize and accelerate training, the affine transformation applies weights and biases to the input, and the activation introduces nonlinearity. This represents a standard layer structure of the ANNs [16]. The final layer of the network omitted a nonlinear activation, instead passing its output to a standard sigmoid function to generate the final outcome. The input dimensions of layers 1 to 4 were 24, *m*_2_, *m*_3_, and *m*_4_, while the output dimensions were *m*_2_, *m*_3_, *m*_4_, and 1, where *m*_2_, *m*_3_, and *m*_4_ are network design parameters that were set between 5 and 50. The first 3 layers used hyperbolic tangent activations. Each model had fewer than 10,000 trainable parameters. These parameters were optimized based on minimizing the binary cross-entropy function, and the Adam optimizer [20] with recommended settings was used for the optimization procedure. Finally, the ANN-GA model, *M*_GA_(*x*), was taken as the average of the 5 models; that is, $M_{GA}\left(x\right):=\frac{1}{5}\sum_{i=1}^{5}M_{i}(x)$. As the values of *M*_GA_(*x*) are always between 0 and 1, the output of *M*_GA_(*x*) is viewed as the predicted probability of belonging to group GA.

**Figure 1.**
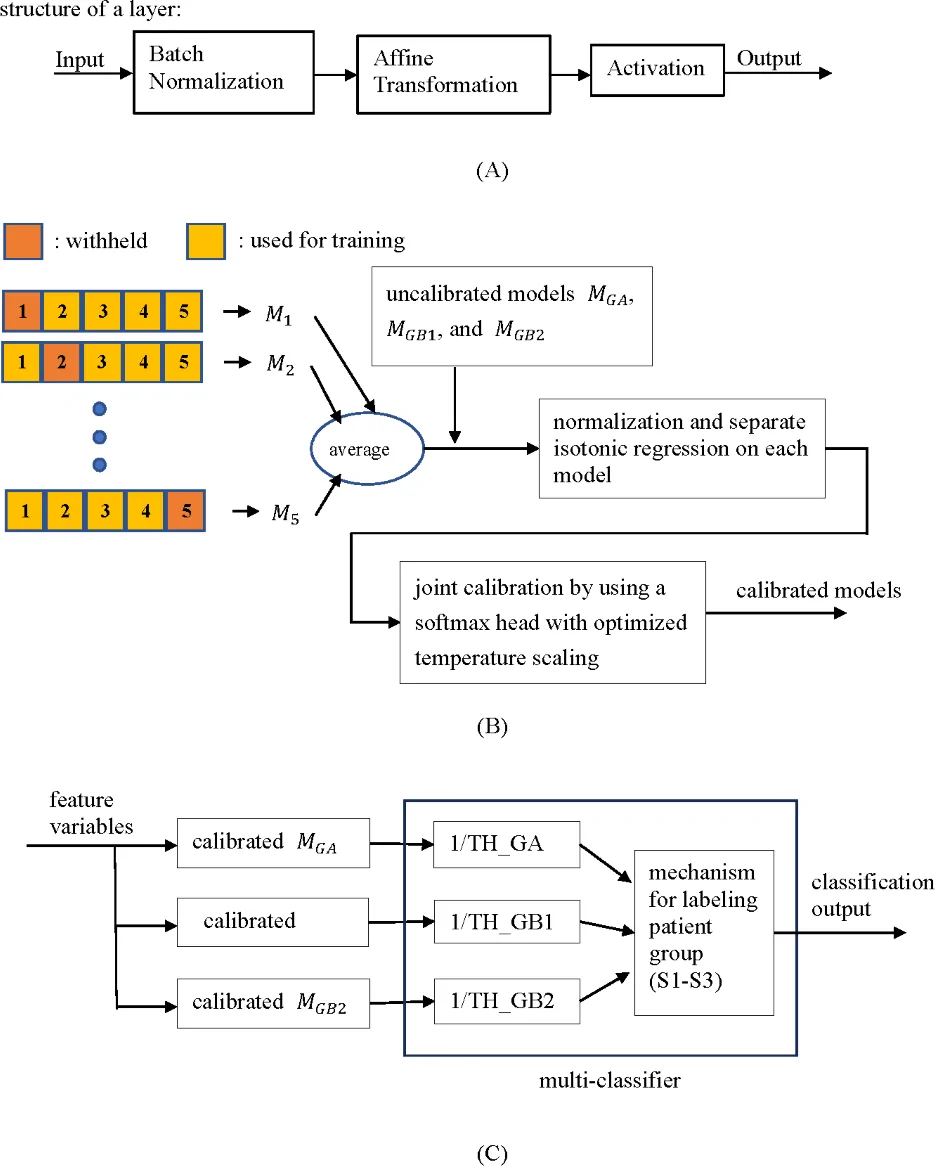
Illustration of the proposed artificial neural network (ANN) models. (A) Illustration of the structure of a layer. (B) Illustration of the construction of the ANN models *M*_GA_, *M*_GB1_, and *M*_GB2_. (C) Illustration of the full model for multiclassification. The blocks “1/TH_GA,” “1/TH_GB1,” and “1/TH_GB2” mean that the input of the block is to be divided by the optimal thresholds of calibrated models ANN-GA, ANN-GB1, and ANN-GB2, respectively. GA: critical patients with acute coronary syndrome; GB1: critical patients without acute coronary syndrome; GB2: low-risk patients.

In the next step, the outputs of the 3 uncalibrated ANN models were jointly normalized so that their predicted probabilities for a given event summed to unity. Each model then underwent separate calibration using isotonic regression [21] to align the individual binary predictions with observed event rates. Because this independent calibration generally breaks the sum-to-unity property, a final softmax function with optimized temperature scaling was applied to the joint postisotonic outputs to restore coherent multiclass probabilities. Specifically, let *p*_A_, *p*_B1_, and *p*_B2_ be the postisotonic probabilities for GA, GB1, and GB2 classification, respectively. In the final step, these probabilities are modified as follows:


(2) $$p_{i}^{\prime }=\frac{e^{\frac{\ln(p_{i})}{T}}}{\sum_{i\in \{A,B1,B2\}}e^{\frac{\ln(p_{i})}{T}}},i\in \{A,B1,B2\}$$

where *T* denotes the temperature scaling factor, which is optimized to minimize the binary cross-entropy loss of the recalibrated probabilities. The optimized temperature scaling factor *T* is 0.935. Figure 1A and B illustrate the layer structure and the flow for constructing the ANN models, including the 2-step calibration pipeline. The hyperparameter configurations used for model training are summarized in Table 1.

To verify the similarity between training and testing datasets, we compared feature distributions by calculating category percentages for categorical variables and means with SDs for quantitative variables. Performance evaluation was conducted based on the 2-step calibrated probabilities obtained by applying (calibrated) models to the testing set, with the area under the receiver operating characteristic curve (AUROC) serving as the primary metric. Optimal classification thresholds were derived from the training set’s receiver operating characteristic (ROC) curves by maximizing the Youden index calculated from the calibrated probabilities [22]. These thresholds were then applied to evaluate model sensitivity (recall), specificity, positive predictive value (PPV, or precision), negative predictive value (NPV), and *F*_1_-score. Precision-recall (PR) curves and the areas under the precision-recall curves (AUPRC) were also analyzed. Finally, to evaluate clinical utility, decision curve analysis was applied to the 2-step calibrated ANN models, benchmarking their performance against traditional “treat-all” and “treat-none” strategies.

Furthermore, to evaluate whether the ANN architecture offers superior predictive power, we benchmarked the ANN models against their logistic regression (LR) counterparts (LR-GA, LR-GB1, and LR-GB2). Note that by restricting function *f_i_*(*x*) to be affine, the model *M_i_*(*x*) becomes an LR model. To ensure a rigorous comparison, the LR models were constructed as ANN models with such a specific architectural constraint, optimized, and calibrated via the same computational process. As their ANN counterparts, the LR models were trained using the same training data by optimizing the same loss function, following the same data imbalance strategies, and using the same hyperparameters for the optimization engine. Furthermore, they were tested on the identical testing datasets and evaluated using the same performance metrics as the ANN models.

Moreover, in order to assess potential performance disparity between different patient groups, subgroup analysis with respect to age, sex, and kidney function was performed. The kidney function was evaluated based on estimated glomerular filtration rate (eGFR), which was calculated using the 2021 Chronic Kidney Disease Epidemiology Collaboration formula [23]. The model’s performance on different subgroups was compared based on their ROC curves and the corresponding AUROCs.

Multiclassifiers were constructed for the final prediction using the 2-step calibrated outputs of the 3 ANN models. Each model output was first divided by the respective optimal threshold for binary classification, yielding the scores *o*_GA_, *o*_GB1_, and *o*_GB2_ for each patient. By dividing the respective optimal threshold, the scores *o*_GA_, *o*_GB1_, and *o*_GB2_ measure a patient’s “distance” to the clinically accepted risk requirement for each classification. Three classification rules were evaluated for making the final classification prediction:

- S1: Assign the patient to the group with the highest score.
- S2: Assign to GA if *o*_GA_≥1; otherwise, to GB2 if *o*_GB2_≥1; remaining patients to GB1.
- S3: Assign to GA if *o*_GA_≥1; otherwise to GB2 if *o*_GB2_≥*o*_GB1_, or to GB1 if *o*_GB2_*<o*_GB1_.

Figure 1C illustrates the multiclassification process. Each multiclassifier was applied to the testing data, and their performance was evaluated by comparing miss rates, sensitivity, specificity, PPV, NPV, and *F*_1_-score.

Finally, we benchmark a multiclassifier based on ANN models with Youden optimal thresholds against that based on the LR models to evaluate the performance gains afforded by the ANN architecture. We further conducted sensitivity analyses by varying the GA-classification threshold, allowing us to explore the inherent trade-off between identifying high-risk ACS (GA) and low-risk (GB2) cases.

**Table 1.** Hyperparameter configurations used for model training^a,b^.

	Nodes (layer 1 to 4)	Learning rate	Batch size	Total epochs	Class weights	
					Positive	Negative
ANN^c^-GA^d^ model	(15, 10, 3, 1)	0.002	8267	2000	9	1
ANN-GB1^e^ model	(15, 10, 3, 1)	0.01	8267	2000	7	1
ANN-GB2^f^ model	(15, 10, 3, 1)	0.01	8267	2000	1	1

a Any parameters not explicitly listed in the table were kept at their default settings as specified by the respective programs.

b Batch normalization (for all models): momentum=0.8; epsilon=0.001. Early stop criteria (for all models): none. Joint postisotonic recalibration using softmax function (for all models): the temperature scaling factor *T*=0.935.

c ANN: artificial neural network.

d GA: critical patients with acute coronary syndrome.

e GB1: critical patients without acute coronary syndrome.

f GB2: low-risk patients.

### Potential Improvement on the Patient Flow

To evaluate the potential of ANN models in reducing ED overcrowding, we analyzed GB2 patient records from the testing dataset, focusing on ED LoS and the number of hs-TnT tests received. While 2 tests are typically required before discharge, some patients were released after 1 test. By identifying such cases, we estimated the average time saved if GB2 patients were discharged earlier based on model predictions, thereby assessing the model’s impact on improving patient flow.

### Importance of Feature Variables and Model Interpretability

To evaluate the contribution of each feature variable to the predictive power of the models, Gini diversity indices [24] were calculated for classifying patients into GA, GB1, and GB2. Variables with notably high importance were selected, and ANN models were trained and calibrated using only these variables or the remaining ones. The performance of these models was compared to that of the full-variable models to assess whether key features alone provided sufficient predictive power. Furthermore, the Shapley Additive Explanations (SHAP) value analysis [25] was conducted on the ANN models to clarify the impact of each feature variable on the model’s output. The results were examined to seek a clinical explanation of the model’s predictive power.

### Statistical Analyses

Statistical analyses were conducted with SPSS for Windows (version 24.0; IBM Corp). Categorical variables were compared with the chi-square test, and continuous variables (means and SD) with the independent 2-tailed *t* test.

### Ethical Considerations

A formal study protocol was not prospectively prepared for this retrospective analysis. Additionally, the study was not registered in a public clinical trial database, as it used existing electronic health records for model development and evaluation. The study was approved by the institutional review board of the National Cheng Kung University Hospital, Tainan, Taiwan (A-ER-111‐199). The study was conducted following the principles set forth in the Declaration of Helsinki. The requirement for informed consent was waived due to the retrospective nature of the study. Study data were deidentified before processing to protect the privacy of the participants. Patients or the public were not involved in the design, conduct, reporting, or dissemination plans of this research. This study was reported according to the TRIPOD+AI (Transparent Reporting of a Multivariable Prediction Model for Individual Prognosis Or Diagnosis Using Artificial Intelligence) guidelines, and the completed checklist is provided in Checklist 1.

## Results

### Characteristics of Study Samples

#### Overview of the Study Population

**Figure 2.**
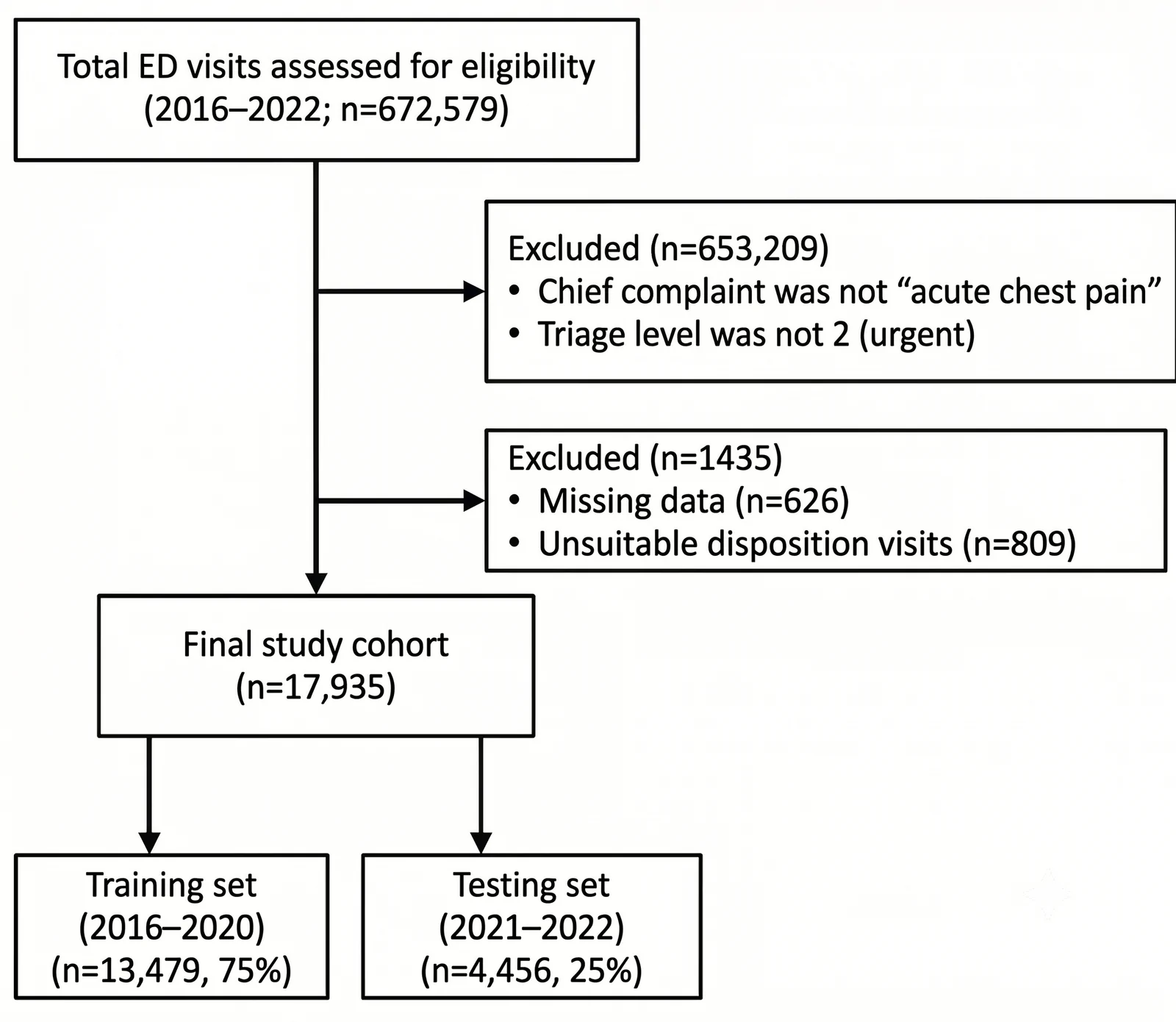
Participant flow diagram detailing the numbers of patients excluded. ED: emergency department.

From the initial 672,579 total visits between January 2016 and December 2022, 653,209 visits were excluded, as they did not meet the inclusion criteria of adult patients presenting with ACP triaged as level 2 (urgent). Of the remaining potential visits, 809 visits were excluded due to unsuitable dispositions (eg, discharge against medical advice and disposition undocumented), while 626 were excluded due to missing triage data, missing examination results, or incomplete medical histories. This resulted in a final study cohort of 17,935 eligible visits. A CONSORT (Consolidated Standards of Reporting Trials)-style patient flow diagram detailing the number of patients excluded is shown in Figure 2.

The total of 17,935 visits were recorded from 13,618 unique patients. Among these, 2384 individuals contributed multiple visits, and 1802 made repeat visits within the same category. Within this latter group, 36 patients made repeat visits in group GA, 321 in group GB1, and 1537 in group GB2, with mean visit frequencies of 2.1 (SD 0.28), 2.3 (SD 0.85), and 2.8 (SD 2.13) per patient, respectively. High-frequency use (over 5 visits) was rare, occurring in only 4 patients in GB1 and 88 in GB2, with no such cases in GA. Of the total visits, 6.74% (1209/17,935) were categorized as GA, 21.59% (3873/17,935) as GB1, and 71.67% (12,853/17,935) as GB2. Furthermore, we identified that 523 patients had separate visits across the training and testing cohorts, with 142 patients (contributing 151 of 4456 visits in the testing dataset) overlapping between the adjacent years of 2020 and 2021. We have reviewed these instances and confirm that they represent unique, independent clinical events separated by significant time intervals; none of them are duplicate records. Furthermore, no short-term revisits (within 72 hours) crossed the temporal split. Because these visits represent different physiological data points, they do not constitute data leakage and were retained to maintain a realistic representation of clinical practice.

Patient characteristics for the training and testing datasets are detailed in Tables S1-S6 in Multimedia Appendix 1. For each patient group, both datasets demonstrated statistical parity across all feature variable distributions. Furthermore, significant differences (*P* value <.01) were observed in many feature variables when comparing each group against all others (eg, GA vs non-GA).

Among the 1435 excluded visits, the average age was 60.6 (SD 17.04) years, 61.67% (885/1453) were male, 38.32% (550/1435) were female, 7.46% (107/1435) are with a history of coronary artery disease (CAD), and 12.13% (174/1435) were with a history of congestive heart failure. These numbers are in line with those of the study cohort.

To further substantiate the clinical grounding of our study labels, we performed a post-hoc analysis of the clinical profiles and follow-up outcomes for both the low-risk surrogate group (GB2) and the non-ACS urgent illness group (GB1).

#### Validation of Using “Discharged After Short Observation” as Low-Risk Surrogate to Define GB2

To validate the clinical safety of the ground-truth labeling for the low-risk group (GB2), we conducted a comprehensive post-hoc analysis of all 12,853 visits in this category. Short-term safety was confirmed by a 72-hour ED revisit rate for chest pain of 2.16% (277/12,853) and a negligible 72-hour hospitalization rate for non-ACS urgent illnesses of 0.05% (7/12,853).

Over a 30-day follow-up period, the cumulative major adverse cardiac event (MACE) rate—comprising ACS and out-of-hospital cardiac arrest (OHCA)—was 0.20% (25/12,853), with a 30-day hospitalization rate of 0.19% (24/12,853). Formal adjudication of these 25 MACE cases identified 21 ACS diagnoses and 4 OHCAs. The 4 OHCAs occurred 7 to 16 days after discharge. The 21 ACS included 7 ST-elevation myocardial infarctions (STEMIs). Notably, all 7 STEMI cases presented 3 to 29 days after discharge and exhibited significant new changes on follow-up ECGs compared to their index visits. These findings suggest that such events likely reflect late disease progression rather than initial diagnostic failure.

Furthermore, we evaluated the risk of missed lethal noncardiac conditions. Among the 897 (6.98%) patients who revisited the ED within 30 days, we identified only 3 cases of pulmonary embolism and 1 aortic dissection (AD). Within the testing cohort specifically, there was only 1 case of pulmonary embolism and no case of AD. This represents a remarkably low combined incidence of 0.03% (4/12,853 total; 1/3237 testing) for these critical events. Collectively, these results substantiate that using “ED discharge” as a surrogate for low-risk status is both safe and clinically robust.

#### Composition of the Non-ACS Urgent Illness Group GB1

The GB1 category was established to encompass acute conditions requiring medical intervention or hospitalization other than ACS. Analysis of the primary diagnoses within this group revealed a diverse spectrum of clinically significant conditions: congestive heart failure (746/3873, 19.26%), acute pulmonary edema (666/3873, 17.20%), cardiac arrhythmia (585/3873, 15.10%), pneumonia (454/3873, 11.72%), AD (194/3873, 5.01%), and pneumothorax (39/3873, 1.01%). Additionally, 28.53% (1105/3873) of cases in GB1 were hospitalized for intensive clinical observation of chest pain. These findings indicate that a broad range of urgent medical states beyond coronary events have been taken into account in the model training process.

### Model Performance

**Figure 3.**
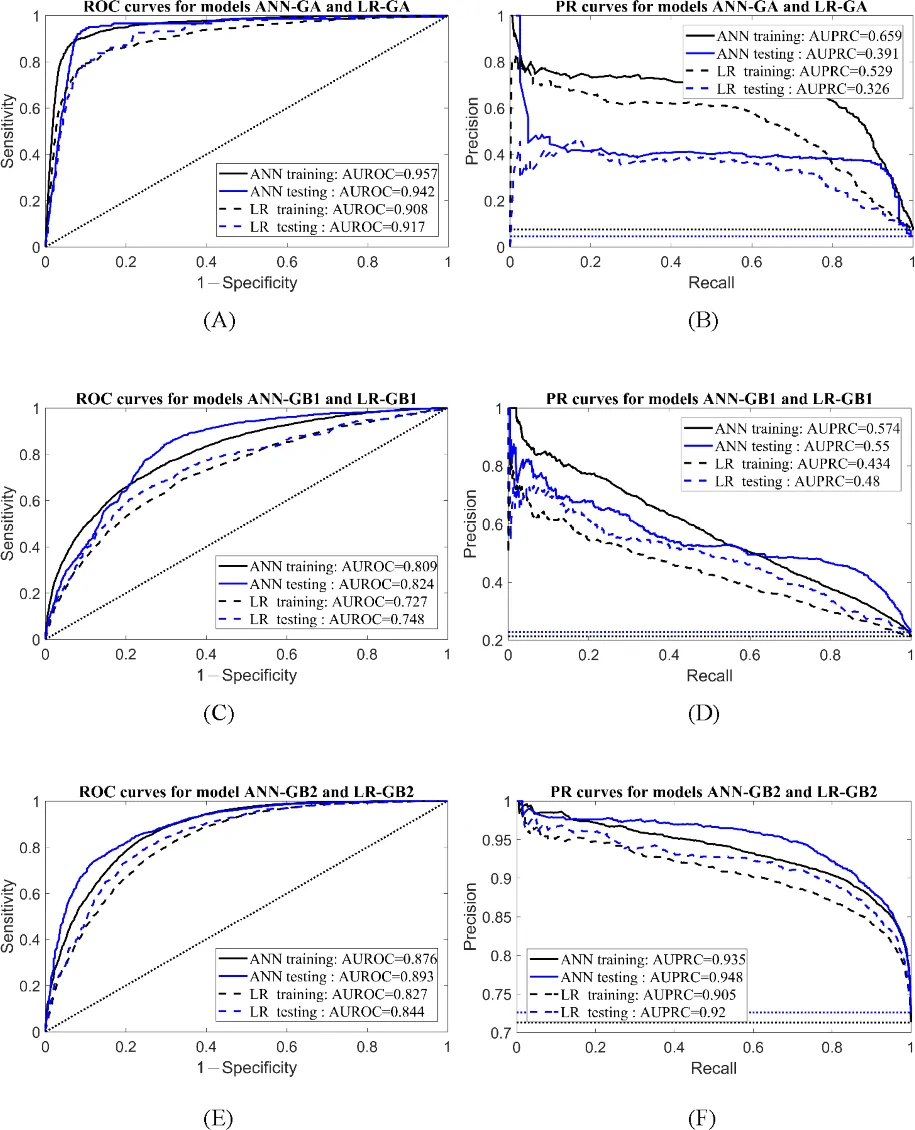
ROC and PR curves of the ANN-GA, ANN-GB1, ANN-GB2 and LR-GA, LR-GB1, LR-GB2 models. (A and B) Curves of ANN-GA and LR-GA models, (C and D) curves of ANN-GB1 and LR-GB1 models, and (E and F) curves of ANN-GB2 and LR-GB2 models. ANN: artificial neural network; AUPRC: area under the precision-recall curve; AUROC: area under the receiver operating characteristic curve; GA: critical patients with acute coronary syndrome; GB1: critical patients without acute coronary syndrome; GB2: low-risk patients; LR: logistic regression; PR: precision-recall; ROC: receiver operating characteristic.

Figure 3 presents the ROC and PR curves of the 3 ANN and the 3 LR models for both training and testing datasets. The full confusion matrices and class-wise performance metrics, including AUROC, accuracy, sensitivity, specificity, PPV, NPV, *F*_1_-score, and AUPRC at respective optimal cutoff thresholds, are summarized in Tables 2 and 3. For multiclass classification, full confusion matrices and performance metrics under 3 decision mechanisms (S1-S3) are summarized in Tables 4 and 5. The ANN-based multiclassifiers (thereafter referred to as ANN-S1, ANN-S2, and ANN-S3) were able to achieve high sensitivity in identifying GA patients and high PPV and high sensitivity in identifying GB2 patients. Notably, ANN-S3 yielded 0.941 sensitivity (95% CI 0.908‐0.973) for GA and 0.837 sensitivity (95% CI 0.824‐0.850) and 0.911 PPV (95% CI 0.900‐0.921) for GB2.

**Table 2.** Confusion matrices of the ANN (artificial neural network)-GA (critical patients with acute coronary syndrome), ANN-GB1 (critical patients without acute coronary syndrome), ANN-GB2 (low-risk patients) and LR-GA, LR-GB1, LR-GB2 models applied to the training and testing datasets^a^.

	Predict 1	Predict 0
ANN-GA, training		
Label 1	901	105
Label 0	969	11,504
ANN-GA, testing		
Label 1	191	12
Label 0	417	3836
ANN-GB1, training		
Label 1	1975	882
Label 0	2419	8203
ANN-GB1, testing		
Label 1	627	389
Label 0	635	2805
ANN-GB2, training		
Label 1	8064	1552
Label 0	933	2930
ANN-GB2, testing		
Label 1	2811	426
Label 0	326	893
LR-GA, training		
Label 1	848	158
Label 0	1820	10,653
LR-GA, testing		
Label 1	164	39
Label 0	506	3747
LR-GB1, training		
Label 1	1973	884
Label 0	3616	7006
LR-GB1, testing		
Label 1	753	263
Label 0	1215	2225
LR-GB2, training		
Label 1	7960	1656
Label 0	1243	2620
LR-GB2, testing		
Label 1	2716	521
Label 0	359	860

a The respective threshold values of each model can be found in Table 3.

**Table 3.** Summary of the performance measures of the ANN (artificial neural network)-GA (critical patients with acute coronary syndrome), ANN-GB1 (critical patients without acute coronary syndrome), ANN-GB2 (low-risk patients) and LR-GA, LR-GB1, LR-GB2 models applied to the training and testing datasets.

Model and dataset	AUROC^a^ (95% CI)	TH^b^	Accuracy (95% CI)	Sensitivity (95% CI)	Specificity (95% CI)	PPV^c^ (95% CI)	NPV^d^ (95% CI)	*F*_1_ (95% CI)	AUPRC^e^ (95% CI)
ANN-GA									
Train	0.957 (0.948‐0.965)	0.0744	0.920 (0.916‐0.925)	0.896 (0.877‐0.915)	0.922 (0.918‐0.927)	0.482 (0.459‐0.504)	0.991 (0.989‐0.993)	0.627 (0.606‐0.647)	0.659 (0.650‐0.668)
Test	0.942 (0.920‐0.965)	0.0744	0.904 (0.895‐0.912)	0.941 (0.908‐0.973)	0.902 (0.893‐0.911)	0.314 (0.277‐0.351)	0.997 (0.995‐0.999)	0.471 (0.428‐0.513)	0.391 (0.368‐0.413)
LR- GA									
Train	0.908 (0.896‐0.921)	0.0553	0.853 (0.847‐0.859)	0.843 (0.820‐0.865)	0.854 (0.848‐0.860)	0.318 (0.300‐0.336)	0.985 (0.983‐0.988)	0.462 (0.442‐0.482)	0.529 (0.517‐0.542)
Test	0.917 (0.891‐0.943)	0.0553	0.878 (0.868‐0.887)	0.808 (0.754‐0.862)	0.881 (0.871‐0.891)	0.245 (0.212‐0.277)	0.990 (0.986‐0.993)	0.376 (0.337‐0.416)	0.326 (0.300‐0.352)
ANN-GB1									
Train	0.809 (0.799‐0.819)	0.2441	0.755 (0.748‐0.762)	0.691 (0.674‐0.708)	0.772 (0.764‐0.780)	0.449 (0.435‐0.464)	0.903 (0.897‐0.909)	0.545 (0.531‐0.559)	0.574 (0.564‐0.584)
Test	0.824 (0.808‐0.841)	0.2441	0.770 (0.758‐0.783)	0.617 (0.587‐0.647)	0.815 (0.802‐0.828)	0.497 (0.469‐0.524)	0.878 (0.867‐0.890)	0.550 (0.525‐0.573)	0.550 (0.534‐0.567)
LR- GB1									
Train	0.727 (0.716‐0.738)	0.1777	0.666 (0.658‐0.674)	0.691 (0.674‐0.708)	0.660 (0.651‐0.669)	0.353 (0.340‐0.366)	0.888 (0.881‐0.895)	0.467 (0.454‐0.480)	0.434 (0.423‐0.445)
Test	0.748 (0.729‐0.766)	0.1777	0.668 (0.654‐0.682)	0.741 (0.714‐0.768)	0.647 (0.631‐0.663)	0.383 (0.361‐0.404)	0.894 (0.882‐0.906)	0.505 (0.483‐0.529)	0.480 (0.462‐0.499)
ANN-GB2									
Train	0.876 (0.870‐0.881)	0.7069	0.816 (0.809‐0.822)	0.839 (0.831‐0.846)	0.758 (0.745‐0.772)	0.896 (0.890‐0.903)	0.654 (0.640‐0.668)	0.866 (0.861‐0.872)	0.935 (0.929‐0.940)
Test	0.893 (0.884‐0.902)	0.7069	0.831 (0.820‐0.842)	0.868 (0.857‐0.880)	0.733 (0.708‐0.757)	0.896 (0.885‐0.907)	0.677 (0.652‐0.702)	0.882 (0.873‐0.891)	0.948 (0.939‐0.957)
LR-GB2									
Train	0.827 (0.820‐0.834)	0.7152	0.785 (0.778‐0.792)	0.828 (0.820‐0.835)	0.678 (0.663‐0.693)	0.865 (0.858‐0.872)	0.613 (0.598‐0.627)	0.846 (0.840‐0.852)	0.905 (0.899‐0.912)
Test	0.844 (0.832‐0.855)	0.7152	0.803 (0.791‐0.814)	0.839 (0.826‐0.852)	0.705 (0.680‐0.731)	0.883 (0.872‐0.895)	0.623 (0.597‐0.648)	0.861 (0.851‐0.870)	0.920 (0.908‐0.931)

a AUROC: area under the receiver operating characteristic curve.

b TH: threshold determined by Youden index.

c PPV: positive predictive value.

d NPV: negative predictive value.

e AUPRC: area under the precision-recall curve.

**Table 4.** Full confusion matrices of the multiclassifiers artificial neural network (ANN)-S1, ANN-S2, and ANN-S3 applied to the testing data^a^.

	Predict GA^b^	Predict GB1^c^	Predict GB2^d^
ANN-S1			
Label GA	187	11	5
Label GB1	297	458	261
Label GB2	63	464	2710
ANN-S2			
Label GA	191	6	6
Label GB1	330	369	317
Label GB2	87	342	2808
ANN-S3			
Label GA	191	7	5
Label GB1	330	425	261
Label GB2	87	441	2709

a The class-wise metrics for labeling GA and GB2 can be found in Table 5.

b GA: critical patients with acute coronary syndrome.

c GB1: critical patients without acute coronary syndrome.

d GB2: low-risk patients.

**Table 5.** Summary of the performance measures of the multiclassifiers S1, S2, and S3 applied to the testing dataset.

Multiclassifier and classification	Miss rate (95% CI)	Sensitivity (95% CI)	Specificity (95% CI)	PPV^a^ (95% CI)	NPV^b^(95% CI)	*F*_1_ (95% CI)
ANN-S1^c^						
GA^d^	0.079 (0.042‐0.116)	0.921 (0.884‐0.958)	0.915 (0.907‐0.924)	0.342 (0.302‐0.382)	0.996 (0.994‐0.998)	0.499 (0.456‐0.542)
GB2^e^	0.163 (0.150‐0.176)	0.837 (0.824‐0.850)	0.782 (0.759‐0.805)	0.911 (0.900‐0.921)	0.644 (0.620‐0.668)	0.872 (0.863‐0.881)
ANN-S2						
GA	0.059 (0.027‐0.092)	0.941 (0.908‐0.973)	0.902 (0.893‐0.911)	0.314 (0.277‐0.351)	0.997 (0.995‐0.999)	0.471 (0.429‐0.512)
GB2	0.133 (0.121‐0.144)	0.867 (0.856‐0.879)	0.735 (0.710‐0.760)	0.897 (0.886‐0.907)	0.676 (0.651‐0.701)	0.882 (0.874‐0.890)
ANN-S3						
GA	0.059 (0.027‐0.092)	0.941 (0.908‐0.973)	0.902 (0.893‐0.911)	0.314 (0.277‐0.351)	0.997 (0.995‐0.999)	0.471 (0.429‐0.512)
GB2	0.163 (0.150‐0.176)	0.837 (0.824‐0.850)	0.782 (0.759‐0.805)	0.911 (0.900‐0.921)	0.643 (0.619‐0.668)	0.872 (0.863‐0.881)

a PPV: positive predictive value.

b NPV: negative predictive value.

c ANN: artificial neural network.

d GA: critical patients with acute coronary syndrome.

e GB2: low-risk patients.

Furthermore, Tables 6 and 7 provide the complete confusion matrices and performance metrics for multiclassifiers using the S3 mechanism. These summaries compare the multiclassifiers based on ANN and LR models (referred to as ANN-S3 and LR-S3, respectively)—both using Youden-optimal thresholds—alongside an ANN variant with a reduced GA classification threshold (referred to as ANN-S3-L). For ANN-S3-L, the GA classification threshold was set at 0.0167 (significantly lower compared to 0.0744 for ANN-S3 and 0.0553 for LR-S3). This value was determined by identifying the maximum threshold at which the ANN-GA model achieved a sensitivity exceeding 0.95 on the training data, a conservative approach intended to prioritize clinical safety and minimize false negatives.

**Table 6.** Full confusion matrices of the multiclassifier S3 on testing data, based on (1) artificial neural network (ANN) models with Youden thresholds (ANN-S3), (2) ANN models with the GA^a^ threshold reduced to 0.0167 (ANN-S3-L), and (3) logistic regression (LR) models with Youden thresholds (LR-S3)^b^.

	Predict GA	Predict GB1^c^	Predict GB2^d^
ANN-S3			
Label GA	191	7	5
Label GB1	330	425	261
Label GB2	87	441	2709
ANN-S3-L			
Label GA	196	4	3
Label GB1	501	291	224
Label GB2	363	326	2548
LR-S3			
Label GA	164	32	7
Label GB1	346	463	207
Label GB2	160	902	2175

a GA: critical patients with acute coronary syndrome.

b The class-wise metrics for labeling GA and GB2 can be found in Table 7.

c GB1: critical patients without acute coronary syndrome.

d GB2: low-risk patients.

**Table 7.** Summary of the performance measures of the multiclassifier S3 (on testing data) based on the 3 sets of binary-classification models.

Multiclassifier and classification	Miss rate (95% CI)	Sensitivity (95% CI)	Specificity (95% CI)	PPV^a^ (95% CI)	NPV^b^ (95% CI)	*F*_1_ (95% CI)
ANN-S3^c^						
GA^d^	0.059 (0.027‐0.092)	0.941 (0.908‐0.973)	0.902 (0.893‐0.911)	0.314 (0.277‐0.351)	0.997 (0.995‐0.999)	0.471 (0.429‐0.512)
GB2^e^	0.163 (0.150‐0.176)	0.837 (0.824‐0.850)	0.782 (0.759‐0.805)	0.911 (0.900‐0.921)	0.643 (0.619‐0.668)	0.872 (0.863‐0.881)
ANN-S3-L						
GA	0.034 (0.009‐0.060)	0.966 (0.940‐0.991)	0.797 (0.785‐0.809)	0.185 (0.162‐0.208)	0.998 (0.996‐0.999)	0.310 (0.278‐0.343)
GB2	0.213 (0.199‐0.227)	0.787 (0.773‐0.801)	0.814 (0.792‐0.836)	0.918 (0.908‐0.928)	0.590 (0.567‐0.614)	0.848 (0.837‐0.858)
LR-S3^f^						
GA	0.192 (0.138‐0.246)	0.808 (0.754‐0.862)	0.881 (0.871‐0.891)	0.245 (0.212‐0.277)	0.990 (0.986‐0.993)	0.376 (0.334‐0.416)
GB2	0.328 (0.312‐0.344)	0.672 (0.656‐0.688)	0.824 (0.803‐0.846)	0.910 (0.899‐0.922)	0.486 (0.465‐0.508)	0.773 (0.761‐0.785)

a PPV: positive predictive value.

b NPV: negative predictive value.

c ANN: artificial neural network.

d GA: critical patients with acute coronary syndrome.

e GB2: low-risk patients.

f LR: logistic regression.

**Figure 4.**
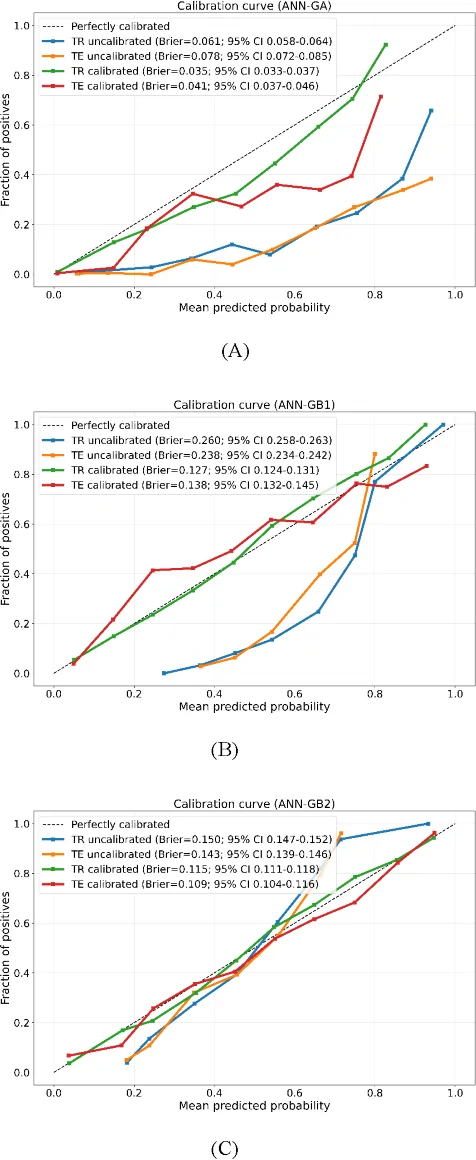
Calibration curves with Brier scores for ANN-GA, ANN-GB1, and ANN-GB2 models on the training and testing datasets. (A) Results for ANN-GA, (B) results for ANN-GB1, and (C) results for ANN-GB2. ANN: artificial neural network; GA: critical patients with acute coronary syndrome; GB1: critical patients without acute coronary syndrome; GB2: low-risk patients; TE: testing; TR: training.

**Figure 5.**
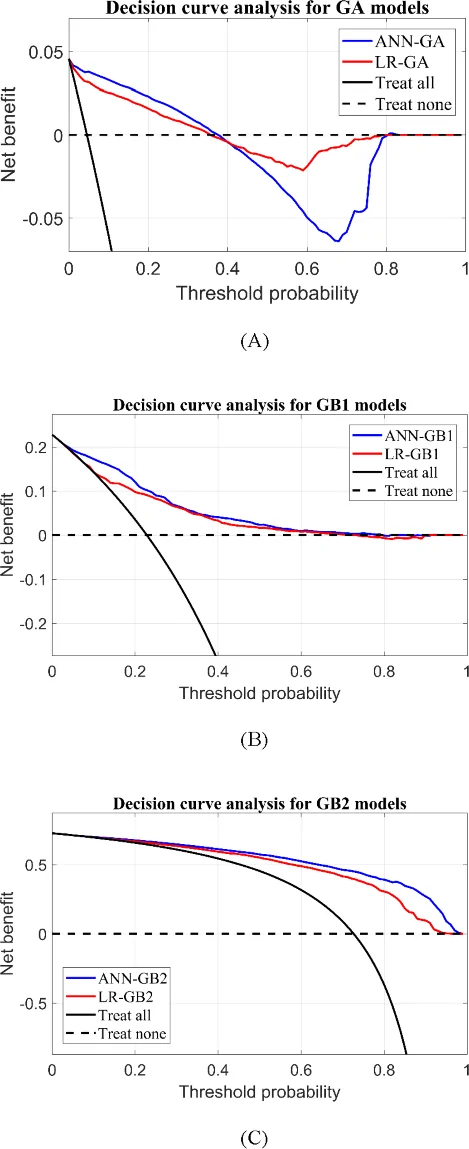
Results of decision curves analysis of ANN and LR models. (A) Decision curves of GA models, (B) decision curves of GB1 models, and (C) decision curves of GB2 models. ANN: artificial neural network; GA: critical patients with acute coronary syndrome; GB1: critical patients without acute coronary syndrome; GB2: low-risk patients; LR: logistic regression.

Calibration curves with Brier scores (95% CIs) for ANN-GA, ANN-GB1, ANN-GB2 models on the training and testing datasets are presented in Figure 4. The results indicate that the calibrated models render predicted probabilities more aligning with the observed risk. ANN-GB2 appears to be very well calibrated on both datasets, while ANN-GB1 somewhat underestimates and ANN-GA overestimates the risk. The results of the decision curve analysis are illustrated in Figure 5. The decision curves show that across a broad range of probability thresholds, all models demonstrated superior clinical utility compared to the traditional “treat-all” and “treat-none” baseline strategies. Notably, for the GB1 and GB2 models, this improved net benefit was maintained across almost the full range of probability thresholds.

**Figure 6.**
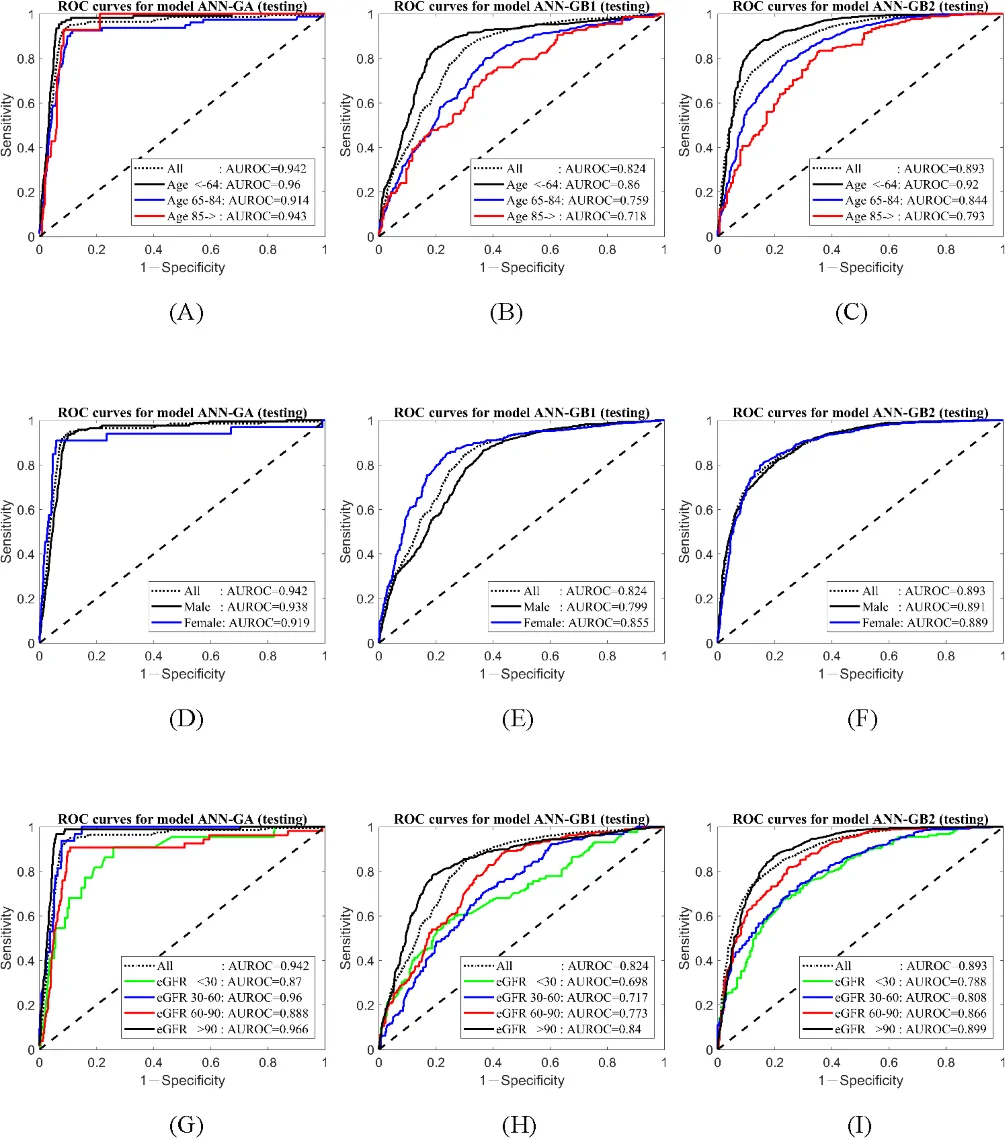
Results of ANN-GA, ANN-GB1, and ANN-GB2 models on different subgroups testing data. (A-C) Comparison among different age groups, (D-F) comparison between male and female sex groups, and (G-I) comparison among different eGFR strata. ANN: artificial neural network; AUROC: area under the receiver operating characteristic curve; eGFR: estimated glomerular filtration rate; GA: critical patients with acute coronary syndrome; GB1: critical patients without acute coronary syndrome; GB2: low-risk patients.

Analysis of subgroup performance was based on the ROC curves of the calibrated ANN models on different subgroups (testing) data. Specifically, we consider 3 age bands (below 65, 65‐85, and above 85 years), male and female sexes, and 4 eGFR strata (below 30, 30‐60, 60‐90, and above 90). The results are presented in Figure 6. Figure 6D-F shows that the ROC curves for male and female subgroups closely align with those of the full dataset, indicating negligible disparity of the model’s predictive power among different sex subgroups. However, notable disparities exist regarding age and eGFR. Specifically, the model underperformed in older-age and low-eGFR subgroups, which exhibited both lower AUROC scores and ROC curves that fell substantially below the aggregate baseline.

### Sensitivity Analysis

To ensure that the 142 patients overlap between the adjacent years of 2020 (belonging to the training cohort) and 2021 (belonging to the testing cohort) did not bias our findings via static baseline variables (eg, age, sex, and comorbidities), a sensitivity analysis was performed by excluding the 151 testing visits by these individuals. The resulting performance metrics remained tightly aligned with the primary analysis. In particular, the AUROC, sensitivity, and PPV for both GA and GB2 classifications remained well within the original 95% CIs. This confirms that cohort overlap did not artificially inflate model performance. A comprehensive comparison is detailed in Tables S7 and S8 in Multimedia Appendix 1.

Furthermore, to evaluate whether the heavy positive skew of the raw hs-TnT data impacted model performance, we performed a sensitivity analysis by retraining and calibrating the ANN models under identical conditions and replacing the raw hs-TnT values with their log-transformed counterparts. Models trained with log-transformed hs-TnT showed slightly lower performance; yet, their results remained tightly aligned with our primary architecture, as reflected by heavily overlapping 95% CIs. Specifically, the training AUROC scores for the primary vs log-transformed (“-T”) models were ANN-GA: 0.957 (95% CI 0.948‐0.965) vs ANN-GA-T: 0.951 (95% CI 0.941‐0.960), ANN-GB1: 0.809 (95% CI 0.799‐0.819) vs ANN-GB1-T: 0.794 (95% CI 0.784‐0.804), and ANN-GB2: 0.876 (95% CI 0.870‐0.881) vs ANN-GB2-T: 0.869 (95% CI 0.864‐0.875). This demonstrates that while batch normalization primarily stabilizes mini-batch activations, the neural network’s architecture was robust to the empirical distribution and skewness of the raw cardiac biomarker data. A comprehensive performance comparison, including AUPRC scores, is provided in Table S9 in Multimedia Appendix 1.

### Potential Improvement of the Patient Flow

In the testing dataset, 3237 patients were categorized as GB2, indicating noncritical conditions. Among them, 3087 had complete data for ED length-of-stay analysis. Of these patients, 1247 received a single hs-TnT test (T1 subgroup, consistent with a single-sample rule-out strategy [26]; median ED LoS 139, IQR 108-195 minutes), whereas 1840 received 2 hs-TnT tests (T2 subgroup, consistent with an every-3-hour serial testing strategy [27]; median ED LoS 285, IQR 251-366 minutes). Based on the clinical variables available at the time of the initial laboratory panel, the ANN-S3 multiclassifier identified 2653 patients as GB2, while 434 GB2 patients were classified as non-GB2.

Among the model-identified GB2 patients, 1170 were from the T1 subgroup, representing 93.83% (1170/1247) of patients who were discharged after a single hs-TnT test in actual clinical practice. This high concordance suggests that the model frequently aligned with physicians’ real-world decisions to manage selected low-risk patients using a single-sample strategy. In addition, 1483 model-identified GB2 patients were from the T2 subgroup, representing 80.60% (1483/1840) of patients who underwent serial hs-TnT testing. These patients represent a retrospectively identified subgroup who might have been considered eligible for a single-sample protocol based on the risk-assessment model, although they were managed with serial testing in actual practice.

**Figure 7.**
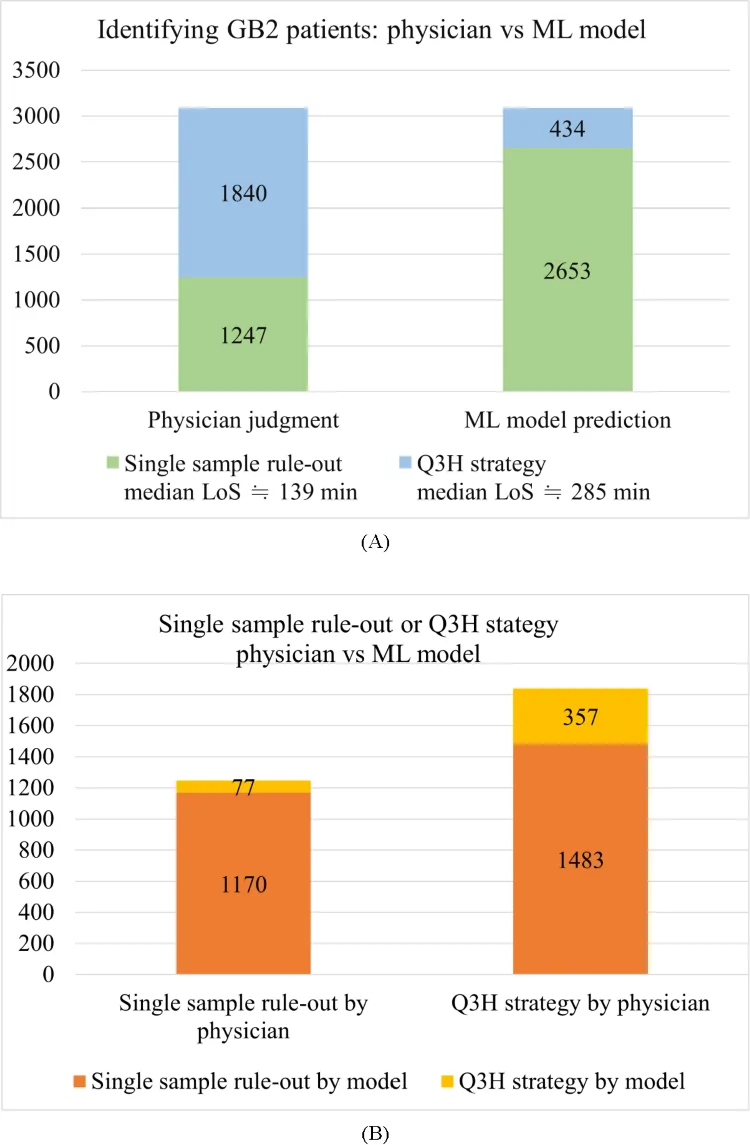
(A) Number of GB2 patients identified based on “single sample rule-out” (green) and “Q3H strategy” (blue) protocols. With the assistance of the ML model, the number of GB2 patients identified by the “single sample rule-out” protocol could be more than doubled compared to the number by physician judgment. (B) Comparison between physician decision and ML model prediction on “single sample rule-out” and “Q3H strategy” protocols. The model highly agreed with the physician decision on “single sample rule-out” (1170/1247 or 93.8%), while in addition correctly identified 1483 more GB2 patients who went through the “Q3H strategy” protocol based on the “single sample rule-out” protocol. GB2: low-risk patients; LoS: length of stay; ML: machine learning; Q3H: every 3 hours.

The longer median LoS observed in the T2 subgroup compared with the T1 subgroup suggests that serial hs-TnT testing was associated with longer ED stays in this retrospective cohort. However, these observational differences should not be interpreted as direct evidence that model implementation would reduce LoS by the same magnitude, because the original decision to perform single versus serial testing was likely influenced by physician-perceived risk and other clinical workflow factors. Therefore, our findings, as illustrated in Figure 7, should be interpreted as demonstrating the model’s potential to enhance ED throughput by identifying additional low-risk candidates for a single-sample strategy, rather than as definitive evidence of actual operational improvement. The actual impact on ED LoS, discharge timing, and patient flow will require prospective evaluation in real-world clinical workflows.

### Importance of Feature Variables and Model Interpretability

For each group classification, the 7 most important feature variables (referred to as “key feature variables”) identified by Gini diversity analysis are as follows:

- For group GA: the 4 blood test results, CAD, and diastolic and systolic blood pressures (BPs).
- For group GB1: the 4 blood test results, CAD, age, and heart rate.
- For group GB2: the 4 blood test results, CAD, age, and systolic BP.

**Figure 8.**
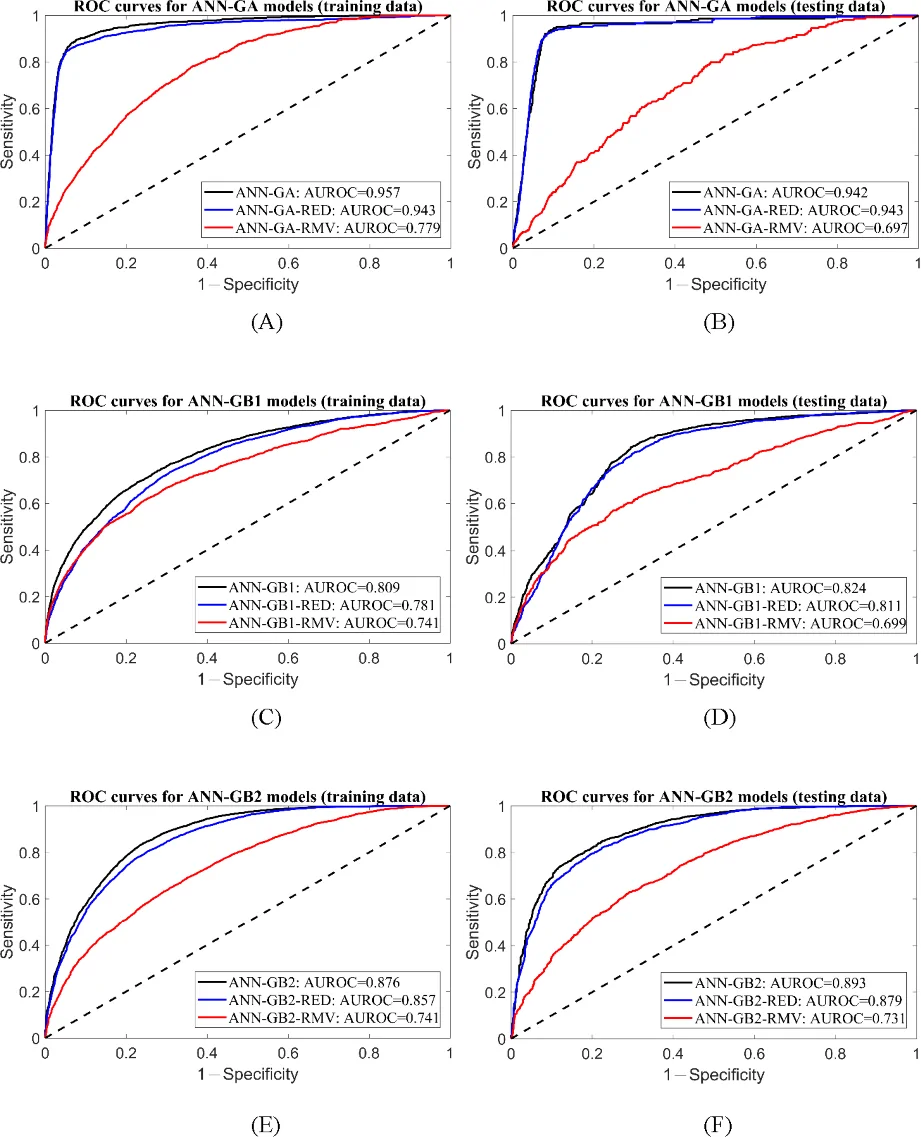
ROC curves of the ANN-GA, ANN-GB1, ANN-GB2 models trained with or with only or without key feature variables. (A and B) Curves of ANN-GA models on training and testing data, (C and D) curves of ANN-GB1 models on training and testing data, and (E and F) curves of ANN-GB2 models on training and testing data. ANN: artificial neural network; AUROC: area under the receiver operating characteristic curve; GA: critical patients with acute coronary syndrome; GB1: critical patients without acute coronary syndrome; GB2: low-risk patients; RED: models trained using only key feature variables; ROC: receiver operating characteristic; RMV: models trained using all but key feature variables.

The ANN models trained using only key feature variables were referred to as ANN-GA-RED, ANN-GB1-RED, and ANN-GB2-RED, respectively. Models trained using all features except the key feature variables were named ANN-GA-RMV, ANN-GB1-RMV, and ANN-GB2-RMV, respectively. Figure 8 displays the ROC curves of these models, along with those of ANN-GA, ANN-GB1, and ANN-GB2. The detailed performance metrics of these models on the testing data are summarized in Table S10 in Multimedia Appendix 1.

**Figure 9.**
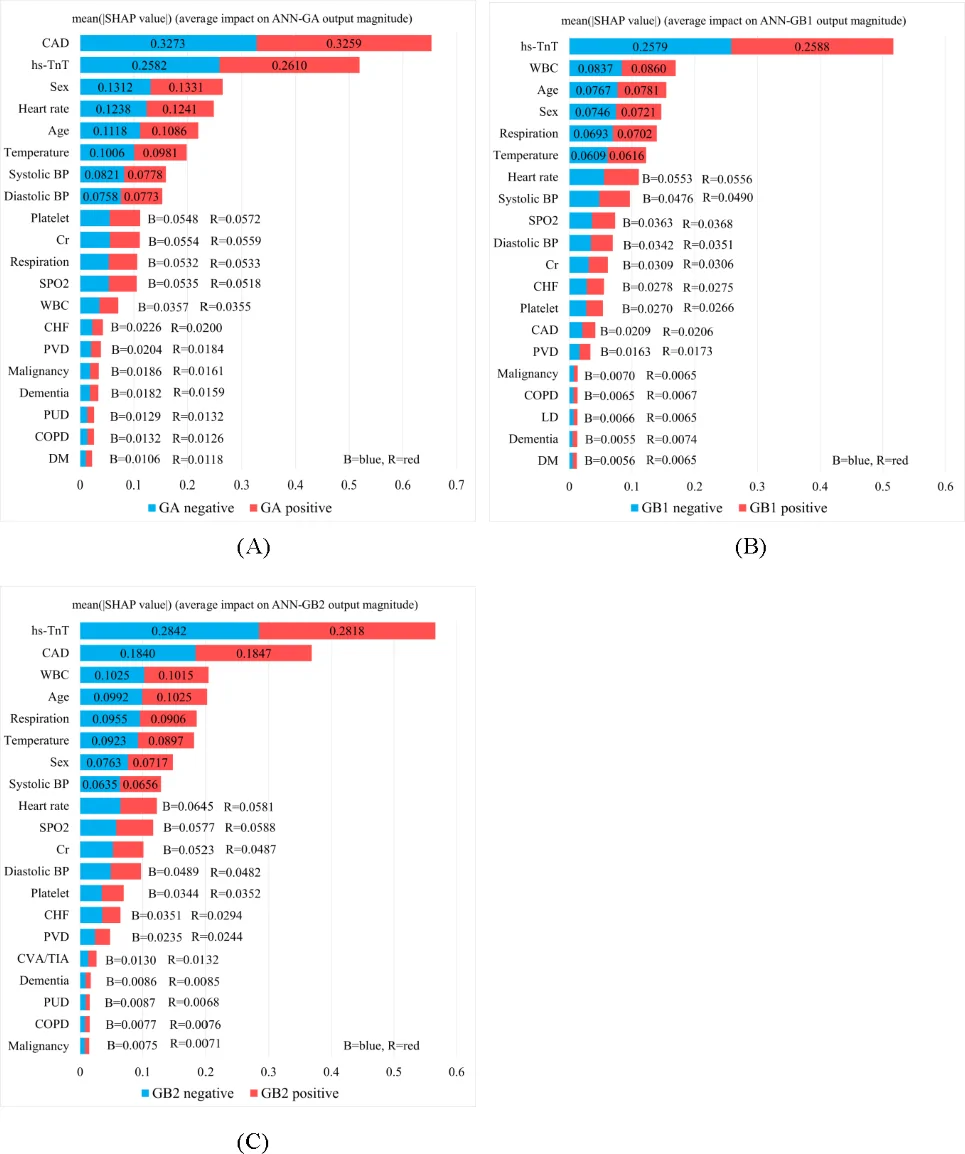
Results of the SHAP value analysis for ANN-GA, ANN-GB1, and ANN-GB2 models. (A) Average impact of each feature variable on the ANN-GA model output, (B) average impact of each feature variable on the ANN-GB1 model output, and (C) average impact of each feature variable on the ANN-GB2 model output. ANN: artificial neural network; BP: blood pressure; CAD: coronary artery disease; CHF: congestive heart failure; COPD: chronic obstructive pulmonary disease; Cr: creatinine; CVA/TIA: cerebrovascular accident/transient ischemic attack; DM: diabetes mellitus; GA: critical patients with acute coronary syndrome; GB1: critical patients without acute coronary syndrome; GB2: low-risk patients; hs-TnT: high-sensitivity cardiac troponin T; LD: liver disease; PUD: peptic ulcer disease; PVD: peripheral vascular disease; SHAP: Shapley Additive Explanations; SPO_2_: peripheral oxygen saturation; WBC: white blood cell.

Regarding the impact of each feature variable on the model’s output, the results of SHAP value analysis are presented in Figure 9. For each model, the top 7 “high impact” feature variables are as follows:

- For model ANN-GA: CAD, hs-TnT, sex, heart rate, age, temperature, and systolic BPs.
- For model ANN-GB1: hs-TnT, white blood cell (WBC), age, sex, respiration, temperature, and heart rate.
- For model ANN-GB2: hs-TnT, CAD, WBC, age, respiration, temperature, and sex.

Apparently, the predicted probabilities rendered by ANN-GA and ANN-GB2 models are driven by 5 common feature variables: CAD, hs-TnT, age, sex, and temperature. In addition, the output of the ANN-GB1 model is predominantly driven by the hs-TnT variable, whose impact is tripled compared to those of other variables.

## Discussion

### Principal Findings

In this study, machine learning models were developed to assist in identifying patients with ACS (GA), those without ACS but with other severe illness (GB1), and low-risk patients (GB2). The individual ANN classification models—ANN-GA, ANN-GB1, and ANN-GB2—all demonstrated impressive performance based on testing AUROC scores: ANN-GA and ANN-GB2 achieved scores close to or greater than 0.9, while ANN-GB1 achieved 0.824. In terms of AUPRC scores, ANN-GB1 and ANN-GB2 achieved 0.55 and 0.948, respectively, indicating a good balance between precision and recall. ANN-GA performed relatively poorly on the AUPRC score, achieving only 0.391. However, this is still a significant improvement compared to a no-skill classifier, for which the AUPRC score would be less than 0.05. The superior predictive powers of the ANN models are evidenced by their significant performance gains over their LR counterparts. This margin is clearly demonstrated by the ROC curves in Figure 3, and the comprehensive metrics are summarized in Tables 2 and 3.

The primary objectives for multiclassification are two-fold: (1) maximizing sensitivity for patients with ACS (GA) due to the life-threatening nature of the condition and (2) ensuring high sensitivity and PPV for low-risk patients (GB2) to streamline the ED patient flow. Among the models evaluated, the ANN-S3 classifier emerged as the superior choice. While ANN-S2 matched its sensitivity for GA, it experienced a lower PPV for GB2 patients. ANN-S1 achieved (almost) identical GB2 performance but exhibited notably lower sensitivity for GA cases. The LR counterpart, LR-S3, proved inferior due to prohibitively low GA and GB2 sensitivities (0.808 and 0.672, respectively). To prioritize clinical safety, the ANN-S3-L variant was developed by lowering the GA classification threshold from 0.0744 to 0.0167. This adjustment resulted in a much higher GA sensitivity (0.966) and NPV (0.998), though it reduced GA specificity from 0.902 to 0.797, as well as GB2 sensitivity from 0.837 to 0.787. This shift ultimately diminished the model’s ability to identify low-risk patients, highlighting the inherent trade-off between clinical safety and operational efficiency.

The S3 multiclassifier uses adjusted scores to differentiate between GB1 (requiring acute care) and GB2 (suitable for discharge after observation) classifications. To prioritize patient safety, the GB1 adjustment factor is nearly 3 times larger than that of GB2 (1/0.2441 vs 1/0.7069). This scaling biases borderline decisions against the GB2 classification, favoring clinical caution over statistical parity. These adjusted scores essentially measure a patient’s “distance” from the clinically accepted risk requirement for respective classification. For example, if a patient’s risk profile exceeds the GB1 threshold (adjusted score>1) but remains below the GB2 threshold (adjusted score<1), the mechanism defaults to GB1. This design is based on the principle that meeting a specific “required” risk level is a more significant clinical milestone than achieving a high raw score that fails to cross a safety threshold. While we acknowledge that this lack of statistical parity may reduce GB2 sensitivity, we view this as a necessary trade-off to enhance the safety of high-risk patients.

Notably, the ANN-based multiclassifier S3 misclassified 5 ACS (GA) cases as “low-risk” (GB2), as shown in Table 6. We have conducted a detailed review of the 5 ACS cases to identify specific failure modes. The misclassified cohort shared a specific “low-risk” profile: (1) a lack of prior CAD history, (2) a relatively young age (median<65 years), (3) hemodynamic and respiratory stability, and (4) initial hs-TnT levels below the 99th percentile URL of 14 ng/L (with 6 of 7 cases<11 ng/L). Identifying ACS in patients who lack traditional risk factors, present as physiologically stable, and maintain “normal” biomarkers represents a significant diagnostic boundary for the model. Such cases remain a formidable challenge even for experienced clinicians [26]. Recognizing these failure patterns is vital for the responsible integration of AI in the ED, ensuring that the model serves as an objective support tool while physician judgment remains the primary safeguard for atypical or early presentations.

Regarding subgroup performance, our analysis revealed that model predictive power decreases in older populations and in patients with lower eGFRs. These results reflect the inherent complexity of diagnosing acute conditions among the older patients in the ED, where they often present with atypical symptoms and multiple comorbidities that obscure traditional clinical signals [27,28]. The observed performance drop suggests that ACS-associated multivariable patterns are significantly more heterogeneous in older cohorts than in younger ones. Moreover, while hs-TnT was a high-impact variable across all classification groups, renal impairment acts as a known confounder. Chronically elevated baseline hs-TnT levels in patients with renal disease complicate the distinction between acute myocardial injury and chronic cardiac strain [29]. Consequently, the diagnostic utility of hs-TnT diminishes as eGFR declines, leading to the observed degradation in model performance. These findings underscore that a “one-size-fits-all” model may be insufficient for high-risk, complex populations. To ensure the responsible deployment of AI in clinical settings, our future work will focus on developing specialized models tailored to the unique physiological and biomarker profiles of older patients and patients who are renally impaired.

Furthermore, the observed performance disparities may stem from the underrepresentation of specific subgroups during training. For instance, patients older than 85 years of age constitute only 7.8% of the training dataset, while those with an eGFR of 30‐60 mL/minute/1.73 m² and below 30 mL/minute/1.73 m² represent just 13% and 11%, respectively. To mitigate this training bias in future iterations, oversampling strategies such as the synthetic minority oversampling technique could be used to better balance these cohorts.

The current 2021 American Heart Association (AHA) and American College of Cardiology (ACC) and 2023 European Society of Cardiology guidelines emphasize serial high-sensitivity troponin testing as the primary standard of care for the evaluation of ACP. Our findings are not intended to supersede these class I recommendations; rather, they explore the potential of AI to support existing “single-sample rule-out” concepts for specific low-risk populations, as discussed in the 2021 AHA and ACC guidelines for patients with prolonged symptom onset. By integrating hs-TnT with 23 other demographic and clinical feature variables, our ANN model offers a multidimensional risk assessment that may help clinicians interpret “gray-zone” troponin levels in older or comorbid patients. This approach serves as a CDS tool, providing an additional layer of evidence to assist physicians in their disposition decisions while remaining within the safety boundaries established by contemporary clinical practice.

While clinical guidelines such as the European Society of Cardiology 0/1 hour or 0/2 hour algorithms are established standards for ruling out ACS, their impact on operational efficiency in real-world practice is often limited. Findings from the European Society of Cardiology 0h/1h Troponin Rule-Out Protocol trial suggest that implementing the 0/1 hour protocol may not significantly reduce ED LoS or increase early discharge rates compared to standard care [30]. This discrepancy exists because actual clinical workflows are fundamentally driven by physician judgment, which synthesizes patient history and multifaceted clinical assessments rather than relying solely on serial laboratory values. Our ANN model addresses this gap by integrating 24 decision variables, including renal function (creatinine), hematological parameters (WBC and platelet counts), vital signs, and extensive medical history. By using this multidimensional approach, the model identifies low-risk features more comprehensively than simple heuristics—such as isolated troponin thresholds—and more closely mirrors the integrative nature of expert clinical reasoning. Furthermore, the objective risk stratification provided by the AI model serves as a reliable “second opinion.” This decision support is particularly valuable in high-pressure ED environments, where it can mitigate decision fatigue and cognitive burden among emergency physicians. By enhancing the confidence of clinicians in early discharge decisions, the model has the potential to optimize patient flow and alleviate ED overcrowding without compromising the established safety standards of current practice.

Our findings suggest that while point-of-care testing is invaluable for rapid, lean evaluations, an AI-augmented strategy may provide a distinct, complementary pathway for deeper risk stratification. Rather than imposing a “one-size-fits-all” mandate, this model offers an objective decision-support tool that reinforces the physician’s initial clinical suspicion. For patients already requiring a standard laboratory workup, the model leverages 24 variables to facilitate a confident, single-sample disposition, which may achieve a substantial reduction in ED LoS compared to traditional serial testing protocols. In our retrospective cohort, patients managed with a single-sample strategy had a median LoS of 139 (IQR 108-195) minutes, compared with 285 (IQR 251-366) minutes among those who underwent serial testing. The difference suggests the potential operational relevance of identifying additional candidates for a single-sample protocol. One should not, however, interpret this difference as a predicted reduction in LoS after model implementation, as the comparison is observational and subject to confounding by indication. Prospective evaluation is required to determine the actual operational impact of a model-driven decision support tool on ED throughput.

The predictive power of each ANN model was primarily driven by a set of key feature variables, as demonstrated by the performance variance in models trained with, with only, and without these variables. These essential features—detailed in the Importance of Feature Variables and Model Interpretability section—encompass patient age, triage blood pressure, prior history of CAD, WBC count, platelet count, serum creatinine, and hs-TnT. The inclusion of age, hypertension, and prior CAD history aligns with traditional cardiovascular risk profiles [31-33]. Models’ reliance on serum creatinine mirrors the documented association between the severity of chronic kidney disease and an increased likelihood of cardiovascular events [34]. Moreover, the predictive weight of WBC and platelet counts reflects the systemic inflammation and platelet activation inherently linked to atherothrombotic processes often observed in patients with ACS [35]. Finally, the inclusion of hs-TnT, the cornerstone biomarker for myocardial injury, reinforces the medical relevance of our feature selection. These key features contribute the primary diagnostic weight. Notably, the “reduced” models—trained exclusively on these high-impact variables—occasionally outperformed the 24-variate “full” models in specific test settings. Furthermore, for categories GB1 and GB2, performance was slightly higher on the testing data than on the training data. This phenomenon likely results from the random fluctuations of low-contributory variables in the training set, which can lead to overfitting. By focusing on high-impact features, the reduced models achieve a form of “implicit regularization,” effectively filtering out stochastic noise. Consequently, these parsimonious models may offer superior operational robustness for real-world clinical deployment, as the marginal utility of the remaining variables is offset by the risk of overfitting to cohort-specific noise.

SHAP value analysis demonstrates high consistency and medical interpretability across both high-risk (GA) and low-risk (GB2) classifications. The most influential variables—specifically hs-TnT, age, sex, and a history of CAD—are firmly grounded in established clinical evidence [36]. In particular, the dominant role of hs-TnT as a fundamental biomarker for myocardial injury aligns perfectly with current diagnostic standards for ACS. Notably, these findings underscore that the model’s predictive power is derived from medically plausible features rather than mere statistical correlation. This alignment between model-identified variables and clinical intuition supports the causal relevance of the model, distinguishing it from conventional “black-box” approaches. Such interpretability is essential for fostering clinician trust and ensuring the responsible integration of AI into medical decision-making.

Most existing studies have focused primarily on the diagnostic performance of AI models in identifying or ruling out ACS [37]. However, relatively few have proposed robust methodologies to evaluate whether the implementation of such models in real-world clinical workflows could yield tangible benefits. In this study, we conducted a retrospective analysis using historical data to quantitatively assess the potential impact of incorporating an AI-based CDS system into the emergency management of patients presenting with ACP. In the high-risk patient subgroup, our model may facilitate earlier recognition of non-STEMI and unstable angina, thus enabling more timely intervention and potentially improving patient outcomes. For low-risk patients, our findings suggest that, compared to physicians’ original clinical decisions, the AI model could safely identify a greater proportion of patients eligible for the single-sample rule-out protocol [38]. This may reduce unnecessary serial blood testing and prolonged ED observation, thereby lowering health care costs and alleviating ED crowding [3].

Furthermore, the integration of AI models into clinical workflow for decision-making support may help mitigate cognitive burden and decision fatigue among emergency physicians, ultimately reducing the risk of burnout [39]. On the other hand, while our ANN model demonstrates high classification performance in a retrospective setting, we acknowledge that significant barriers remain before it can be integrated into routine emergency care. These include sociotechnical challenges such as clinician acceptance, workflow integration, and the medical-legal implications of deviating from serial troponin testing protocols. Our findings should be interpreted as a preliminary step in exploring AI-augmented risk stratification. Future research must focus on prospective validation and implementation science to ensure that such models provide tangible clinical benefits without compromising patient safety or increasing the cognitive load on ED staff.

To ensure the safe and responsible implementation of our model, we emphasize that the AI system is designed as a CDS tool intended to function within a human-AI team framework. Rather than replacing physician judgment, the model provides context-specific risk assessments that emergency physicians can integrate into their clinical workflow. For high-risk cases (GA and GB1), the system can actively alert clinicians, whereas for low-risk patients, it serves as a passive supportive resource. To mitigate risks such as overreliance or user error, clinicians are provided with the basis for model outputs and informed of the system’s limitations. Furthermore, in alignment with Good Machine Learning Practice principles, we acknowledge that the performance of deployed models must be subject to ongoing monitoring in “real-world” use. Our planned future work includes establishing a dedicated monitoring platform to detect dataset drift or performance degradation over time. This framework will include strict controls for retraining and model updates to manage risks such as overfitting or unintended bias, ensuring the model’s safety and effectiveness throughout its total product life cycle.

### Limitation

This study has several limitations. First, this study’s single-center, retrospective design inherently limits the geographic and ethnic generalizability of our findings. We acknowledge that some key baseline characteristics—such as cardiovascular risk profiles, BMI thresholds, and renal function—vary significantly across diverse populations and health care systems. Consequently, the model’s performance may reflect our specific local cohort and might not be immediately applicable to other clinical settings. External validation using larger, multicenter datasets is a mandatory prerequisite before this model can be considered for universal clinical implementation.

Second, our study used ED disposition as the primary surrogate for the “low-risk” (GB2) ground truth, which may not capture adverse events occurring outside the hospital. Although we confirmed the 30-day survival of 8556 patients through subsequent clinical records, remaining patients had no further follow-up in our ED, limiting our ability to definitively confirm their survival status. This is again a characteristic limitation of single-center retrospective designs. However, given the negligible 30-day MACE rate of 0.2%, the GB2 label remains a clinically reliable benchmark for training and evaluating our AI models.

Third, the absence of symptom-onset timing in our dataset represents a significant constraint. The 2021 AHA and ACC Chest Pain Guidelines mandate that single-sample rule-out strategies apply only to patients presenting more than 3 hours after symptoms begin. Since our dataset lacks this temporal data, the model’s clinical use should be limited to late-presenting cohorts to maintain diagnostic safety. Future research will focus on safely extending the model’s utility to early presenters while preserving a high NPV.

Fourth, a notable limitation of this study is that the model does not use raw ECG waveforms, structured ECG interpretations, or qualitative clinical descriptors (eg, specific chest pain characteristics) as direct inputs. These variables are core components of established risk stratification tools such as the History, ECG, Age, Risk factors, Troponin score, or Emergency Department Assessment of Chest Pain Score; their absence in a structured format within our retrospective dataset precluded a direct comparison with these traditional heuristics. However, because our unselected study population included all eligible ED visits regardless of ECG presentation, and since our ground-truth labels were derived from final clinical diagnoses—which inherently incorporate ECG and semiological findings—the model effectively learned to correlate its 24 clinical variables with outcomes already influenced by these factors. While our SHAP analysis highlights the importance of traditional risk factors, the ANN architecture was specifically leveraged to capture complex, multidimensional interactions across the available parameters. Currently, the model is intended to augment, rather than replace, initial physician ECG screening. Future prospective work aims to integrate digitized ECG signal analysis and granular clinical descriptors to further strengthen diagnostic safety and benchmark the model’s additive value against established clinical scores.

Fifth, this model may be subject to spectrum bias, as the inclusion criteria were limited to patients triaged as level-2 (urgent). While this prioritized a high-acuity cohort with the most immediate clinical need, it excludes patients undertriaged to lower levels who may still harbor high-risk pathology. This reliance on triage levels partly stems from a lack of granular, structured chest pain data—such as pain radiation, quality, or alleviating factors—which was primarily captured as unstructured narrative text. Although our current variables align with the standard of care, the absence of these qualitative descriptors might lead to the omission of atypical presentations. Future integration of natural language processing to extract clinical features from nursing notes could mitigate this bias, allowing for a broader patient spectrum and improved differentiation between ACS and other critical conditions across all triage categories.

Sixth, a potential selection bias exists due to the exclusion of 1435 visits with missing or indeterminate data, such as incomplete triage records, examination results, or medical histories. While our analysis shows that the final cohort of 17,935 visits remains highly representative of our institution’s urgent chest pain population, these exclusions may slightly impact the generalizability of our findings. The potential bias can be mitigated when the models are retrained with more available data in the future. Moreover, to minimize predictive uncertainty, we deliberately avoided data imputation, ensuring that no prediction is rendered unless all required feature values are present to inform the physician’s judgment. The system was designed to issue error or warning messages when it encounters missing data or unstructured entries that do not meet the input specifications. However, we recognize that this “complete-case” requirement may limit the real-world utility of our models in clinical settings with less structured data entry. To address this, future iterations will explore multivariate imputation by chained equations, enabling the model to have robust performance in the presence of missing data and mitigating current utility restrictions while maintaining diagnostic reliability.

Seventh, the inferred time savings associated with the model are based on a retrospective comparison of median LoS. These findings are subject to confounding by indication, as the single-test cohort likely represented patients with lower perceived clinical risk. While the results suggest a trend toward improved efficiency through reduced repeat testing, they should be interpreted as the theoretical potential of a single-sample strategy. The actual impact on LoS in prospective deployment may vary depending on clinical judgment, ED crowding, and institutional protocols [40], and cannot be definitively predicted from this observational data.

Eighth, the assessment of potential improvements in ED workflow following model implementation was based on retrospective data. Future prospective studies, ideally incorporating collaboration with experts in operations research, are needed to evaluate the real-world impact of integrating the model into clinical practice.

Finally, the testing AUPRC for the high-risk (GA) group was only 0.391. Given the GA prevalence of only 4.6% in our test cohort, this performance represents a significant improvement over random classification; however, it also reflects a lower PR balance for this rare but critical class. While our model prioritizes a high NPV (>99.5%) to ensure patient safety, this emphasis may result in a higher rate of false positives. This conservative bias could potentially lead to increased resource use and diagnostic observation in the ED. Future work will focus on incorporating dynamic clinical features to enhance precision without compromising the model’s high safety threshold.

### Conclusions

The AI models developed in this study serve as a preliminary proof-of-concept for providing CDS to physicians managing ACP. The multiclassification model we developed achieved 0.941 sensitivity for identifying patients with ACS and 0.837 sensitivity and 0.911 PPV for identifying nonsevere patients. Despite many limitations, these results suggest that integrating AI-based multiclassification algorithms into ED workflows has the potential to enhance risk stratification and optimize resource use. However, we recognize that translating these findings into real-world practice involves significant sociotechnical, medical-legal, and workflow challenges. Ultimately, the objective of this work is to develop tools for supporting ED physicians to accurately evaluate ACP within a broad range of clinical guidelines. To enhance predictive performance and broaden model utility, future iterations will focus on four key areas: (1) retraining models using symptom-onset timing and high-impact features, which may include descriptors extracted from digitized ECG signals and nursing notes; (2) integrating data imputation strategies, such as multivariate imputation by chained equations, to handle missing data; (3) developing dedicated models tailored to specific biomarker profiles, such as patients with low eGFR due to renal failure; and (4) validating the models using larger, diverse, multicenter datasets.

## Supplementary material

10.2196/83099 Multimedia Appendix 1 Descriptive analyses, model performance evaluations, and additional validation analyses.

10.2196/83099 Checklist 1 TRIPOD_AI checklist.

## Abbreviations

ACC: American College of Cardiology
ACP: acute chest pain
ACS: acute coronary syndrome
AD: aortic dissection
AHA: American Heart Association
ANN: artificial neural network
AUPRC: area under the precision-recall curve
AUROC: area under the receiver operating characteristic curve
BP: blood pressure
CAD: coronary artery disease
CDS: clinical decision support
CONSORT: Consolidated Standards of Reporting Trials
ECG: electrocardiogram
ED: emergency department
eGFR: estimated glomerular filtration rate
hs-TnT: high-sensitivity cardiac troponin T
ICD-10: International Classification of Diseases, 10th Revision
LoS: length of stay
LR: logistic regression
MACE: major adverse cardiac event
MI: myocardial infarction
NPV: negative predictive value
OHCA: out-of-hospital cardiac arrest
PPV: positive predictive value
PR: precision-recall
ROC: receiver operating characteristic
SHAP: Shapley Additive Explanations
STEMI: ST-elevation myocardial infarction
TRIPOD+AI: Transparent Reporting of a Multivariable Prediction Model for Individual Prognosis Or Diagnosis Using Artificial Intelligence
WBC: white blood cell
