# Supplementary material for: Patient Stratification for Improving Acute Chest Pain Management and Mitigating Emergency Department Crowding: Machine Learning Model Development and Validation
Source: JMIR Med Inform. 2026 Aug 21;14:e83099. doi: 10.2196/83099 (PMC13496927; doi:10.2196/83099)
Supplement: Supplementary file 2 [file medinform-v14-e83099-s001.docx]

Table S1: Descriptive analysis of the training and testing datasets for GA v.s. Non-GA

|  | **Training set (75.2%**) | | |  | | **Testing set (24.8%)** | | | |  |
| --- | --- | --- | --- | --- | --- | --- | --- | --- | --- | --- |
|  | in GA | not in GA | Total | | P-value | | in GA | not in GA | Total | P-value |
|  | Nr. (%) | Nr. (%) | Nr. (%) | |  | | Nr. (%) | Nr. (%) | Nr. (%) |  |
|  | 1006 (7.46) | 12473 (92.54) | 13479 (100) | |  | | 203(4.56) | 4253 (95.44) | 4456 (100) |  |
| **Sex** |  |  |  | | <.001 | |  |  |  | <.001 |
| Male | 769 (76.44) | 7254 (58.16) | 8023 (59.52) | |  | | 170 (83.74) | 2510 (59.02) | 2680 (60.14) |  |
| Female | 237 (23.56) | 5219 (41.84) | 5456 (40.48) | |  | | 33 (16.26) | 1743 (40.98) | 1776 (39.86) |  |
| **Med Cond** |  |  |  | |  | |  |  |  |  |
| CAD |  |  |  | | <.001 | |  |  |  | <.001 |
| With | 796 (79.13) | 507(4.06) | 1303 (9.67) | |  | | 174 (85.71) | 275 (6.47) | 449 (10.08) |  |
| Without | 210 (20.87) | 11966 (95.94) | 12176 (90.33) | |  | | 29 (14.29) | 3978 (93.53) | 4007 (89.92) |  |
| CTD |  |  |  | | 0.354 | |  |  |  | 0.471 |
| With | 7 (0.70) | 124 (0.99) | 131 (0.97) | |  | | 4 (1.97) | 58 (1.36) | 62 (1.39) |  |
| Without | 999 (99.30) | 12349 (99.01) | 13348 (99.03) | |  | | 199 (98.03) | 4195 (98.64) | 4394 (98.61) |  |
| PVD |  |  |  | | 0.005 | |  |  |  | 0.696 |
| With | 13 (1.29) | 349 (2.80) | 362 (2.69) | |  | | 4 (1.97.) | 102 (2.40) | 106 (2.38) |  |
| Without | 993 (98.71) | 12124 (97.20) | 13117 (97.31) | |  | | 199 (98.03) | 4151 (97.60) | 4350 (97.62) |  |
| PUD |  |  |  | | <.001 | |  |  |  | 0.509 |
| With | 3 (0.30) | 243 (1.95) | 246 (1.83) | |  | | 3 (1.48) | 92 (2.16) | 95 (2.13) |  |
| Without | 1003 (99.70) | 12230 (98.05) | 13233 (98.17) | |  | | 200 (98.52) | 4161 (97.84) | 4361 (97.87) |  |
| LD |  |  |  | | 0.178 | |  |  |  | 0.466 |
| With | 7 (0.70) | 145 (1.16) | 152 (1.13) | |  | | 1 (0.49) | 43 (1.01) | 44 (0.99) |  |
| Without | 999 (99.30) | 12328 (98.84) | 13327 (98.87) | |  | | 202 (99.51) | 4210 (98.99) | 4412 (99.01) |  |
| COPD |  |  |  | | 0.001 | |  |  |  | 0.089 |
| With | 27 (2.68) | 612 (4.91) | 639 (4.74) | |  | | 4 (1.97) | 190 (4.47) | 194 (4.35) |  |
| Without | 979 (97.32) | 11861 (95.09) | 12840 (95.26) | |  | | 199 (98.03) | 4063 (95.53) | 4262 (95.65) |  |
| DM |  |  |  | | <.001 | |  |  |  | 0.005 |
| With | 313 (31.11) | 3115 (24.97) | 3428 (25.43) | |  | | 66 (32.51) | 1015 (23.87) | 1081 (24.26) |  |
| Without | 693 (68.89) | 9358 (75.03) | 10051 (74.57) | |  | | 137 (67.49) | 3238 (76.13) | 3375 (75.74) |  |
| Malignancy |  |  |  | | <.001 | |  |  |  | 0.052 |
| With | 57 (5.67) | 1225 (9.82) | 1282 (9.51) | |  | | 12 (5.91) | 429 (10.09) | 441 (9.90) |  |
| Without | 949 (94.33) | 11248 (90.18) | 12197 (90.49) | |  | | 191 (94.09) | 3824 (89.91) | 4015 (90.10) |  |
| AIDS |  |  |  | | 0.215 | |  |  |  | 0.080 |
| With | 0 (0) | 19 (0.15) | 19 (0.14) | |  | | 2 (0.99) | 12 (0.28) | 14 (0.31) |  |
| Without | 1006 (100) | 12454 (99.85) | 13460 (99.86) | |  | | 201 (99.01) | 4241 (99.72) | 4442 (99.69) |  |
| CHF |  |  |  | | 0.029 | |  |  |  | 0.475 |
| With | 96 (9.54) | 1476 (11.83) | 1572(11.66) | |  | | 15 (7.39) | 376 (8.84) | 391 (8.77) |  |
| Without | 910 (90.46) | 10997 (88.17) | 11907 (88.34) | |  | | 188(92.61) | 3877 (91.16) | 4065 (91.23) |  |
| CVA or TIA |  |  |  | | 0.926 | |  |  |  | 0.758 |
| With | 51 (5.07) | 624 (5.00) | 675 (5.01) | |  | | 11 (5.42) | 210 (4.94) | 221 (4.96) |  |
| Without | 955 (94.93) | 11849 (95.00) | 12804 (94.99) | |  | | 192 (94.58) | 4043 (95.06) | 4235 (95.04) |  |
| Dementia |  |  |  | | 0.369 | |  |  |  | 0.648 |
| With | 6 (0.60) | 108 (0.87) | 114 (0.85) | |  | | 3 (1.48) | 48 (1.13) | 51 (1.14) |  |
| Without | 1000 (99.40) | 12365 (99.13) | 13365 (99.15) | |  | | 200 (98.52) | 4205 (98.87) | 4405 (98.86) |  |

Table S2: Descriptive analysis of the training and testing datasets for GB1 v.s. Non-GB1

|  | **Training set (75.2%**) | | |  | | | **Testing set (24.8%)** | | | | | |  | |  |
| --- | --- | --- | --- | --- | --- | --- | --- | --- | --- | --- | --- | --- | --- | --- | --- |
|  | in GB1 | not in GB1 | Total | | P-value | | | in GB1 | not in GB1 | | Total | | P-value | |  |
|  | Nr. (%) | Nr. (%) | Nr. (%) | |  | | | Nr. (%) | Nr. (%) | | Nr. (%) | |  | |  |
|  | 2857 (21.20) | 10622 (78.80) | 13479 (100) | |  | | | 1016 (22.80) | 3440 (77.20) | | 4456 (100) | |  | |  |
| **Sex** |  |  |  | | | <.001 |  | | |  | |  | | <0.01 | |
| Male | 1891 (66.19) | 6132 (57.73) | 8023 (59.52) | | |  | 683 (67.22) | | | 1997 (58.05) | | 2680 (60.14) | |  | |
| Female | 966 (33.81) | 4490 (42.27) | 5456 (40.48) | | |  | 333 (32.78) | | | 1443 (41.95) | | 1776 (39.86) | |  | |
| **Med Cond** |  |  |  | | |  |  | | |  | |  | |  | |
| CAD |  |  |  | | | <.001 |  | | |  | |  | | <.001 | |
| With | 428 (14.98) | 875 (8.24) | 1303 (9.67) | | |  | 251 (24.70) | | | 198 (5.76) | | 449 (10.08) | |  | |
| Without | 2429 (85.02) | 9747 (91.76) | 12176 (90.33) | | |  | 765 (75.30) | | | 3242 (94.24) | | 4007 (89.92) | |  | |
| CTD |  |  |  | | | <.001 |  | | |  | |  | | 0.792 | |
| With | 44 (1.54) | 87 (0.82) | 131 (0.97) | | |  | 15 (1.48) | | | 47 (1.37) | | 62 (1.39) | |  | |
| Without | 2813 (98.46) | 10535 (99.18) | 13348 (99.03) | | |  | 1001 (98.52) | | | 3393 (98.63) | | 4394 (98.61) | |  | |
| PVD |  |  |  | | | <.001 |  | | |  | |  | | <.001 | |
| With | 171 (5.99) | 191 (1.80) | 362 (2.69) | | |  | 57 (5.61) | | | 49 (1.42) | | 106 (2.38) | |  | |
| Without | 2686 (94.01) | 10431 (98.20) | 13117 (97.31) | | |  | 959 (94.39) | | | 3391 (98.58) | | 4350 (97.62) | |  | |
| PUD |  |  |  | | | <.001 |  | | |  | |  | | 0.117 | |
| With | 76 (2.66) | 170 (1.60) | 246 (1.83) | | |  | 28 (2.76) | | | 67 (1.95) | | 95 (2.13) | |  | |
| Without | 2781 (97.33) | 10452 (98.40) | 13233 (98.17) | | |  | 988 (97.24) | | | 3373 (98.05) | | 4361 (97.87) | |  | |
| LD |  |  |  | | | 0.003 |  | | |  | |  | | 0.012 | |
| With | 47 (1.65) | 105 (0.99) | 152 (1.13) | | |  | 17 (1.67) | | | 27 (0.78) | | 44 (0.99) | |  | |
| Without | 2810 (98.35) | 10517 (99.01) | 13327 (98.87) | | |  | 999 (98.33) | | | 3413 (99.22) | | 4412 (99.01) | |  | |
| COPD |  |  |  | | | <.001 |  | | |  | |  | | 0.404 | |
| With | 184 (6.44) | 455 (4.28) | 639 (4.74) | | |  | 49 (4.82) | | | 145 (4.22) | | 194 (4.35) | |  | |
| Without | 2673 (93.56) | 10167 (95.72) | 12840 (95.26) | | |  | 967 (95.18) | | | 3295 (95.78) | | 4262 (95.65) | |  | |
| DM |  |  |  | | | <.001 |  | | |  | |  | | <.001 | |
| With | 962 (33.67) | 2466 (23.22) | 3428 (25.43) | | |  | 366 (36.02) | | | 715 (20.78) | | 1081 (24.26) | |  | |
| Without | 1895 (66.33) | 8156 (76.78) | 10051 (74.57) | | |  | 650 (63.98) | | | 2725 (79.22) | | 3375 (75.74) | |  | |
| Malignancy |  |  |  | | | <.001 |  | | |  | |  | | <.001 | |
| With | 360 (12.60) | 922 (8.68) | 1282 (9.51) | | |  | 143 (14.07) | | | 298 (8.66) | | 441 (9.90) | |  | |
| Without | 2497 (87.40) | 9700 (91.32) | 12197 (90.49) | | |  | 873 (85.93) | | | 3142 (91.34) | | 4015 (90.10) | |  | |
| AIDS |  |  |  | | | 0.268 |  | | |  | |  | | 0.902 | |
| With | 6 (0.21) | 13 (0.12) | 19 (0.14) | | |  | 3 (0.30) | | | 11 (0.32) | | 14 (0.31) | |  | |
| Without | 2851 (99.79) | 10609 (99.88) | 13460 (99.86) | | |  | 1013 (99.70) | | | 3429 (99.68) | | 4442 (99.69) | |  | |
| CHF |  |  |  | | | <.001 |  | | |  | |  | | <.001 | |
| With | 743 (26.01) | 829 (7.80) | 1572(11.66) | | |  | 197 (19.39) | | | 194 (5.64) | | 391 (8.77) | |  | |
| Without | 2114 (73.99) | 9793 (92.20) | 11907 (88.34) | | |  | 819 (80.61) | | | 3246 (94.36) | | 4065 (91.23) | |  | |
| CVA or TIA |  |  |  | | | <.001 |  | | |  | |  | | <.001 | |
| With | 198 (6.93) | 477 (4.49) | 675 (5.01) | | |  | 75 (7.38) | | | 146 (4.24) | | 221 (4.96) | |  | |
| Without | 2659 (93.07) | 10145 (95.51) | 12804 (94.99) | | |  | 941 (92.62) | | | 3294 (95.76) | | 4235 (95.04) | |  | |
| Dementia |  |  |  | | | 0.514 |  | | |  | |  | | 0.258 | |
| With | 27 (0.95) | 87 (0.82) | 114 (0.85) | | |  | 15 (1.48) | | | 36 (1.05) | | 51 (1.14) | |  | |
| Without | 2830 (99.05) | 10535 (99.18) | 13365 (99.15) | | |  | 1001 (98.52) | | | 3404 (98.95) | | 4405 (98.86) | |  | |

Table S3: Descriptive analysis of the training and testing datasets for GB2 v.s. Non-GB2

|  | **Training set (75.2%**) | | |  | | **Testing set (24.8%)** | | | |  | |  |
| --- | --- | --- | --- | --- | --- | --- | --- | --- | --- | --- | --- | --- |
|  | in GB2 | not in GB2 | Total | P-value | | in GB2 | not in GB2 | Total | | P-value | |  |
|  | Nr. (%) | Nr. (%) | Nr. (%) |  | | Nr. (%) | Nr. (%) | Nr. (%) | |  | |  |
|  | 9616 (71.34) | 3863 (28.66) | 13479 (100) |  | | 3237 (72.64) | 1219 (27.36) | 4456 (100) | |  | |  |
| **Sex** |  |  |  | | <.001 |  |  | |  | | <0.001 | |
| Male | 5363 (55.77) | 2660 (68.86) | 8023 (59.52) | |  | 1827 (56.44) | 853 (69.98) | | 2680 (60.14) | |  | |
| Female | 4253 (44.23) | 1203 (31.14) | 5456 (40.48) | |  | 1410 (43.56) | 366 (30.02) | | 1776 (39.86) | |  | |
| **Med Cond** |  |  |  | |  |  |  | |  | |  | |
| CAD |  |  |  | | <.001 |  |  | |  | | <.001 | |
| With | 79 (0.82) | 1224 (31.69) | 1303 (9.67) | |  | 24 (0.74) | 425 (34.86) | | 449 (10.08) | |  | |
| Without | 9537 (99.18) | 2639 (68.31) | 12176 (90.33) | |  | 3213 (99.26) | 794 (65.14) | | 4007 (89.92) | |  | |
| CTD |  |  |  | | 0.009 |  |  | |  | | 0.559 | |
| With | 80 (0.83) | 51 (1.32) | 131 (0.97) | |  | 43 (1.33) | 19 (1.56) | | 62 (1.39) | |  | |
| Without | 9536 (99.17) | 3812 (98.68) | 13348 (99.03) | |  | 3194 (98.77) | 1200 (98.44) | | 4394 (98.61) | |  | |
| PVD |  |  |  | | <.001 |  |  | |  | | <.001 | |
| With | 178 (1.85) | 184 (4.76) | 362 (2.69) | |  | 45 (1.39) | 61 (5.0) | | 106 (2.38) | |  | |
| Without | 9438 (98.15) | 3679 (95.24) | 13117 (97.31) | |  | 3192 (98.61) | 1158 (95.0) | | 4350 (97.62) | |  | |
| PUD |  |  |  | | 0.227 |  |  | |  | | 0.244 | |
| With | 167 (1.74) | 79 (2.05) | 246 (1.83) | |  | 64 (1.98) | 31 (2.54) | | 95 (2.13) | |  | |
| Without | 9449 (98.26) | 3784 (97.95) | 13233 (98.17) | |  | 3173 (98.02) | 1188 (97.46) | | 4361 (97.87) | |  | |
| LD |  |  |  | | 0.060 |  |  | |  | | 0.043 | |
| With | 98 (1.02) | 54 (1.40) | 152 (1.13) | |  | 26 (0.80) | 18 (1.48) | | 44 (0.99) | |  | |
| Without | 9518 (98.98) | 3809 (98.60) | 13327 (98.87) | |  | 3211 (99.20) | 1201 (98.52) | | 4412 (99.01) | |  | |
| COPD |  |  |  | | 0.012 |  |  | |  | | 0.991 | |
| With | 428 (4.45) | 211 (5.46) | 639 (4.74) | |  | 141 (4.36) | 53 (4.35) | | 194 (4.35) | |  | |
| Without | 9188 (95.55) | 3652 (94.54) | 12840 (95.26) | |  | 3096 (95.64) | 1166 (95.65) | | 4262 (95.65) | |  | |
| DM |  |  |  | | <.001 |  |  | |  | | <.001 | |
| With | 2153 (22.39) | 1275 (33.01) | 3428 (25.43) | |  | 649 (20.05) | 432 (35.44) | | 1081 (24.26) | |  | |
| Without | 7463 (77.61) | 2588 (66.99) | 10051 (74.57) | |  | 2588 (79.95) | 787 (64.56) | | 3375 (75.74) | |  | |
| Malignancy |  |  |  | | 0.001 |  |  | |  | | <.001 | |
| With | 865 (9.0) | 417 (10.79) | 1282 (9.51) | |  | 86 (8.84) | 155 (12.72) | | 441 (9.90) | |  | |
| Without | 8751 (91.0) | 3446 (89.21) | 12197 (90.49) | |  | 2951 (91.16) | 1064 (87.28) | | 4015 (90.10) | |  | |
| AIDS |  |  |  | | 0.778 |  |  | |  | | 0.482 | |
| With | 13 (0.14) | 6 (0.16) | 19 (0.14) | |  | 9 (0.28) | 5 (0.41) | | 14 (0.31) | |  | |
| Without | 9603 (99.86) | 3857 (99.84) | 13460 (99.86) | |  | 3228 (99.72) | 1214 (99.59) | | 4442 (99.69) | |  | |
| CHF |  |  |  | | <.001 |  |  | |  | | <.001 | |
| With | 733 (7.62) | 839 (21.72) | 1572(11.66) | |  | 179 (5.53) | 212 (17.39) | | 391 (8.77) | |  | |
| Without | 8883 (92.38) | 3024 (78.28) | 11907 (88.34) | |  | 3058 (94.47) | 1007 (82.61) | | 4065 (91.23) | |  | |
| CVA or TIA |  |  |  | | <.001 |  |  | |  | | <.001 | |
| With | 426 (4.43) | 249 (6.45) | 675 (5.01) | |  | 135 (4.17) | 86 (7.05) | | 221 (4.96) | |  | |
| Without | 9190 (95.57) | 3614 (93.55) | 12804 (94.99) | |  | 3102 (95.83) | 1133 (92.95) | | 4235 (95.04) | |  | |
| Dementia |  |  |  | | 0.946 |  |  | |  | | 0.201 | |
| With | 81 (0.84) | 33 (0.85) | 114 (0.85) | |  | 33 (1.02) | 18 (1.48) | | 51 (1.14) | |  | |
| Without | 9535 (99.16) | 3830 (99.15) | 13365 (99.15) | |  | 3204 (98.98) | 1201 (98.52) | | 4405 (98.86) | |  | |

Abbreviations: Med Cond, past medical condition; CAD, coronary artery disease; CTD, connective tissue disease; PVD, peripheral vascular disease; PUD, peptic ulcer disease; LD, liver disease; COPD, chronic obstructive pulmonary disease; DM, diabetes mellitus; AIDS, acquired immunodeficiency syndrome; CHF, congestive heart failure; CVA, cerebrovascular accident; TIA, transient ischemic attack.

Table S4: Characteristics of age, vital signs taken at triage, and blood tests of patients in the training and testing datasets. Analysis was conducted for GA v.s. Non-GA

|  | **Training set (75.2%**) | | | **Testing set (24.8%)** | | |
| --- | --- | --- | --- | --- | --- | --- |
|  | in GA | not in GA |  | in GA | not in GA |  |
|  | Mean (SD)  (95% CI) | Mean (SD)  (95% CI) | P-value | Mean (SD)  (95% CI) | Mean (SD)  (95% CI) | P-value |
| **Age** | 65.55 (13.58)  (64.71-66.39) | 62.53 (16.59)  (62.24-62.82) | <.001 | 64.18 (13.83)  (62.28-66.08) | 60.29 (18.19)  (59.74-60.84) | <.001 |
| **Vital Signs** |  |  |  |  |  |  |
| Temperature | 36.39 (0.64)  (36.35-36.43) | 36.59 (0.64)  (36.58-36.60) | <.001 | 36.30 (0.65)  (36.21-36.39) | 36.54 (0.68)  (36.52-36.56) | <.001 |
| Heart rate | 83.19 (20.85)  (81.91-84.48) | 85.65 (19.84)  (85.30-86.00) | <.001 | 83.67 (21.81)  (80.67-86.67) | 86.11 (20.14)  (85.50-86.72) | 0.120 |
| Resp. rate | 19.94 (3.74)  (19.71-20.17) | 19.64 (3.15)  (19.59-19.70) | 0.016 | 19.93 (4.31)  (19.34-20.52) | 19.98 (2.95)  (19.89-20.07) | 0.869 |
| Systolic BP | 142.00 (30.82)  (140.09-143.90) | 144.92 (27.11)  (144.44-145.40) | 0.004 | 140.29 (30.25)  (136.13-144.45) | 142.77 (25.76)  (142.00-143.54) | 0.251 |
| Diastolic BP | 86.57 (20.53)  (85.30-87.84) | 85.26 (17.85)  (84.95-85.57) | 0.050 | 84.28 (20.53)  (81.46-87.10) | 80.15 (17.49)  (79.62-80.68) | 0.005 |
| SpO2 | 89.41 (26.70)  (87.76-91.06) | 90.19 (26.64)  (89.72-90.65) | 0.377 | 95.37 (16.92)  (93.04-97.70) | 95.81 (15.21)  (95.35-96.27) | 0.718 |
| **Blood Tests** |  |  |  |  |  |  |
| hs-TnT | 456.49 (1237.45)  (380.02-532.95) | 50.69 (333.27)  (44.84-56.54) | <.001 | 576.92(1752.96)  (335.77-810.07) | 66.21(532.23)  (50.21-82.21) | <.001 |
| CR | 1.52 (1.81)  (1.41-1.63) | 1.37 (1.78)  (1.34-1.40) | 0.012 | 1.47 (1.89)  (1.21-1.73) | 1.17 (1.68)  (1.12-1.22) | 0.028 |
| Platelet | 223.42 (74.87)  (218.80-228.05) | 226.80 (81.24)  (225.37-228.22) | 0.172 | 229.94 (84.97)  (218.25-241.63) | 226.02 (87.22)  (223.40-228.64) | 0.522 |
| WBC | 9.84 (3.48)  (9.62-10.05) | 8.16 (4.22)  (8.09-8.24) | <.001 | 9.39 (3.96)  (8.85-9.93) | 7.81 (3.84)  (7.69-7.93) | <.001 |

Table S5: Characteristics of age, vital signs taken at triage, and blood tests of patients in the training and testing datasets. Analysis was conducted for GB1 v.s. Non-GB1

|  | **Training set (75.2%**) | | | **Testing set (24.8%)** | | |
| --- | --- | --- | --- | --- | --- | --- |
|  | in GB1 | not in GB1 |  | in GB1 | not in GB1 |  |
|  | Mean (SD)  (95% CI) | Mean (SD)  (95% CI) | P-value | Mean (SD)  (95% CI) | Mean (SD)  (95% CI) | P-value |
| **Age** | 67.83 (15.06)  (67.28-68.38) | 61.39 (16.48)  (61.08-61.70) | <.001 | 68.01 (14.99)  (67.09-68.93) | 58.24 (18.25)  (57.63-58.85) | <.001 |
| **Vital Signs** |  |  |  |  |  |  |
| Temperature | 36.65 (0.79) (36.62-36.68) | 36.55 (0.60)  (36.54-36.56) | <.001 | 36.52 (0.80)  (36.47-36.57) | 36.53 (0.63)  (36.51-36.55) | 0.761 |
| Heart rate | 88.17 (22.91)  (87.33-89.01) | 84.74 (18.98)  (84.38-85.10) | <.001 | 87.98 (24.90)  (86.45-89.51) | 85.42 (18.58)  (84.80-86.04) | 0.002 |
| Resp. rate | 20.65 (4.40)  (20.49-20.81) | 19.40 (2.73)  (19.35-19.45) | <.001 | 20.90 (3.45)  (20.69-21.11) | 19.71 (2.83)  (19.62-19.80) | <.001 |
| Systolic BP | 141.23 (29.94)  (140.13-142.33) | 145.63 (26.61)  (145.12-146.14) | <.001 | 140.62 (28.79)  (138.85-142.39) | 143.26 (25.07)  (142.42-144.10) | 0.008 |
| Diastolic BP | 82.43 (20.37)  (81.68-83.18) | 86.15 (17.31)  (85.82-86.48) | <.001 | 78.58 (21.26)  (77.27-79.89) | 80.86 (16.41)  (80.31-81.41) | 0.002 |
| SpO2 | 90.46 (24.06)  (89.58-91.34) | 90.04 (27.30)  (89.52-90.56) | 0.419 | 95.36 (13.67)  (94.52-96.20) | 95.92 (15.74)  (95.39-96.45) | 0.266 |
| **Blood Tests** |  |  |  |  |  |  |
| hs-TnT | 146.31 (658.29)  (122.17-170.45) | 63.41 (414.46)  (55.53-71.29) | <.001 | 210.27 (1052.54)  (145.55-274.99) | 53.79 (461.38)  (38.37-69.21) | <.001 |
| CR | 2.02 (2.51)  (1.93-2.11) | 1.21 (1.48)  (1.18-1.24) | <.001 | 1.81 (2.78)  (1.64-1.98) | 1.00 (1.14)  (0.96-1.04) | <.001 |
| Platelet | 222.23 (94.24)  (218.77-225.69) | 227.71 (76.73)  (226.25-229.17) | 0.004 | 220.55 (122.37)  (213.03-228.07) | 227.87 (73.48)  (225.41-230.33) | 0.071 |
| WBC | 9.55 (7.07)  (9.29-9.81) | 7.95 (2.88)  (7.90-8.01) | <.001 | 9.39 (6.20)  (9.01-9.77) | 7.44 (2.66)  (7.35-7.53) | <.001 |

Table S6: Characteristics of age, vital signs taken at triage, and blood tests of patients in the training and testing datasets. Analysis was conducted for GB2 v.s. Non-GB2

|  | **Training set (75.2%**) | | | **Testing set (24.8%)** | | |
| --- | --- | --- | --- | --- | --- | --- |
|  | in GB2 | not in GB2 |  | in GB2 | not in GB2 |  |
|  | Mean (SD)  (95% CI) | Mean (SD)  (95% CI) | P-value | Mean (SD)  (95% CI) | Mean (SD)  (95% CI) | P-value |
| **Age** | 60.95 (16.69)  (60.62-61.28) | 67.24 (14.72)  (66.77-67.70) | <.001 | 57.87 (18.43)  (57.24-58.50) | 67.37 (14.87)  (66.53-68.20) | <.001 |
| **Vital Signs** |  |  |  |  |  |  |
| Temperature | 36.57 (0.59)  (36.56-36.58) | 36.58 (0.76)  (36.56-36.60) | 0.270 | 36.54 (0.63)  (36.52-36.56) | 36.48 (0.78)  (36.44-36.52) | 0.018 |
| Heart Rate | 84.91 (18.77)  (84.53-85.28) | 86.87 (22.50)  (86.16-87.58) | <.001 | 85.53 (18.36)  (84.90-86.16) | 87.26 (24.46)  (85.89-88.63) | 0.025 |
| Resp Rate | 19.35 (2.59)  (19.30-19.40) | 20.47 (4.25)  (20.34-20.60) | <.001 | 19.69 (2.71)  (19.60-19.78) | 20.74 (3.62)  (20.54-20.94) | <.001 |
| Systolic BP | 146.02 (26.11)  (145.50-146.54) | 141.43 (30.17)  (140.48-142.38) | <.001 | 143.45 (24.70)  (142.60-144.30) | 140.56 (29.02)  (138.93-142.19) | 0.002 |
| Diastolic BP | 86.10 (16.93)  (85.76-86.44) | 83.51 (20.49)  (82.86-84.16) | <.001 | 80.65 (16.09)  (80.10-81.20) | 79.53 (21.24)  (78.34-80.72) | 0.095 |
| SpO2 | 90.10 (27.36)  (89.56-90.65) | 90.19 (24.78)  (89.41-90.97) | 0.864 | 95.95 (15.67)  (95.41-96.49) | 95.36 (14.26)  (94.56-96.16) | 0.227 |
| **Blood Tests** |  |  |  |  |  |  |
| hs-TnT | 22.28(108.77)  (20.11-24.45) | 227.08 (858.77)  (200.00-254.16) | <.001 | 20.99(127.14)  (16.61-25.37) | 271.33 (1204.78)  (203.70-338.96) | <.001 |
| CR | 1.17 (1.44) (1.14-1.20) | 1.89 (2.36) (1.82-1.96) | <.001 | 0.98 (1.07) (0.94-1.02) | 1.75 (2.65) (1.60-1.90) | <.001 |
| Platelet | 228.15 (76.91) (226.62-229.69) | 222.54 (89.59) (219.71-225.37) | <.001 | 227.74 (72.71) (225.24-230.24) | 222.12 (117.00) (215.55-228.69) | 0.117 |
| WBC | 7.75 (2.74) (7.70-7.80) | 9.62 (6.33) (9.42-9.82) | <.001 | 7.32 (2.51) (7.23-7.41) | 9.39 (5.88) (9.06-9.72) | <.001 |

Abbreviations: SD, standard deviation; CI, confidence interval; BP, blood pressure; Resp, respiratory; SpO2, peripheral oxygen saturation; hs-TnT, high-sensitivity troponin T; CR, creatinine; WBC, white blood cell.

Table S7

Comparison of model performance (ANN-GA, ANN-GB1, and ANN-GB2) on all testing data versus a reduced dataset removing 151 visits from 142 overlapping patients between the adjacent years 2020 and 2021. Abbreviations: ALL, all testing data; RED, testing data with 151 visits removed.

| Model | Data Set | AUROC  (95% CI) | TH | Accuracy  (95% CI) | Sensitivity  (95% CI) | Specificity  (95% CI) | PPV  (95% CI) | NPV  (95% CI) | F1  (95% CI) | AUPRC  (95% CI) |
| --- | --- | --- | --- | --- | --- | --- | --- | --- | --- | --- |
| ANN-GA | ALL | 0.942  (0.920-0.965) | 0.0744 | 0.904  (0.895-0.912) | 0.941  (0.902-0.970) | 0.902  (0.893-0.911) | 0.314  (0.277-0.351) | 0.997  (0.995-0.999) | 0.471  (0.428-0.513) | 0.391  (0.368-0.413) |
|  | RED | 0.942  (0.919-0.964) | 0.0744 | 0.902  (0.893-0.911) | 0.941  (0.908-0.973) | 0.900  (0.891-0.909) | 0.318  (0.281-0.356) | 0.997  (0.995-0.999) | 0.476  (0.432-0.523) | 0.393  (0.371-0.416) |
| ANN-GB1 | ALL | 0.824  (0.808-0.841) | 0.2441 | 0.770  (0.758-0.783) | 0.617  (0.587-0.647) | 0.815  (0.802-0.828) | 0.497  (0.469-0.524) | 0.878  (0.867-0.890) | 0.550  (0.525-0.573) | 0.550  (0.534-0.567) |
|  | RED | 0.826  (0.809-0.842) | 0.2441 | 0.771  (0.759-0.784) | 0.615  (0.585-0.646) | 0.817  (0.804-0.830) | 0.499  (0.471-0.527) | 0.878  (0.866-0.889) | 0.551  (0.526-0.576) | 0.548  (0.532-0.565) |
| ANN-GB2 | ALL | 0.893  (0.884- 0.902) | 0.7069 | 0.831  (0.820-0.842) | 0.868  (0.857-0.880) | 0.733  (0.708-0.757) | 0.896  (0.885-0.907) | 0.677  (0.652-0.702) | 0.882  (0.873-0.891) | 0.948  (0.939-0.957) |
|  | RED | 0.896  (0.887- 0.905) | 0.7069 | 0.832  (0.821-0.843) | 0.870  (0.858-0.881) | 0.734  (0.709-0.759) | 0.896  (0.885-0.907) | 0.681  (0.656-0.707) | 0.883  (0.873-0.892) | 0.950  (0.941-0.959) |

Table S8

Performance comparison of multi-classification model ANN-S3 on all testing data versus a reduced dataset removing 151 visits from overlapping patients.

| Model and Data Set | Classification | Miss Rate  (95% CI) | Sensitivity  (95% CI) | Specificity  (95% CI) | PPV  (95% CI) | NPV  (95% CI) | F1  (95% CI) |
| --- | --- | --- | --- | --- | --- | --- | --- |
| ANN-S3 on ALL | GA | 0.059  (0.027-0.092) | 0.941  (0.908-0.973) | 0.902  (0.893-0.911) | 0.314  (0.277-0.351) | 0.997  (0.995-0.999) | 0.471  (0.429-0.512) |
|  | GB2 | 0.163  (0.150-0.176) | 0.837  (0.824-0.850) | 0.782  (0.759-0.805) | 0.911  (0.900-0.921) | 0.643  (0.619-0.668) | 0.872  (0.863-0.881) |
| ANN-S3 on RED | GA | 0.059  (0.027-0.092) | 0.941  (0.908-0.973) | 0.900  (0.891-0.909) | 0.318  (0.281-0.356) | 0.997  (0.995-0.999) | 0.476  (0.432-0.523) |
|  | GB2 | 0.160  (0.147-0.173) | 0.840  (0.827-0.853) | 0.784  (0.761-0.807) | 0.911  (0.901-0.921) | 0.651  (0.626-0.675) | 0.874  (0.865-0.884) |

Table S9

Performance comparison of the primary ANN models (ANN-GA, ANN-GB1, ANN-GB2) trained with raw hs-TnT values versus the transformed models (ANN-GA-T, ANN-GB1-T, ANN-GB2-T) trained with log-transformed hs-TnT values.

| Model | Data Set | AUROC  (95% CI) | TH | Accuracy  (95% CI) | Sensitivity  (95% CI) | Specificity  (95% CI) | PPV  (95% CI) | NPV  (95% CI) | F1  (95% CI) | AUPRC  (95% CI) |
| --- | --- | --- | --- | --- | --- | --- | --- | --- | --- | --- |
| ANN-GA | Train | 0.957  (0.948-0.965) | 0.0744 | 0.920  (0.916-0.925) | 0.896  (0.877-0.915) | 0.922  (0.918-0.927) | 0.482  (0.459-0.504) | 0.991  (0.989-0.993) | 0.627  (0.606-0.647) | 0.659  (0.650-0.668) |
|  | Test | 0.942  (0.920-0.965) | 0.0744 | 0.904  (0.895-0.912) | 0.941  (0.902-0.970) | 0.902  (0.893-0.911) | 0.314  (0.277-0.351) | 0.997  (0.995-0.999) | 0.471  (0.428-0.513) | 0.391  (0.368-0.413) |
| ANN- GA-T | Train | 0.951  (0.941-0.960) | 0.1038 | 0.926  (0.922-0.930) | 0.874  (0.853-0.894) | 0.930  (0.926-0.935) | 0.502  (0.479-0.526) | 0.989  (0.987-0.991) | 0.638  (0.616-0.658) | 0.659  (0.650-0.668) |
|  | Test | 0.941  (0.919-0.964) | 0.1038 | 0.910  (0.901-0.918) | 0.921  (0.884-0.958) | 0.909  (0.900-0.918) | 0.326  (0.287-0.364) | 0.996  (0.994-0.998) | 0.481  (0.436-0.526) | 0.350  (0.328-0.373) |
| ANN-GB1 | Train | 0.809  (0.799-0.819) | 0.2441 | 0.755  (0.748-0.762) | 0.691  (0.674-0.708) | 0.772  (0.764-0.780) | 0.449  (0.435-0.464) | 0.903  (0.897-0.909) | 0.545  (0.531-0.559) | 0.574  (0.564-0.584) |
|  | Test | 0.824  (0.808-0.841) | 0.2441 | 0.770  (0.758-0.783) | 0.617  (0.587-0.647) | 0.815  (0.802-0.828) | 0.497  (0.469-0.524) | 0.878  (0.867-0.890) | 0.550  (0.525-0.573) | 0.550  (0.534-0.567) |
| ANN-GB1-T | Train | 0.794  (0.784-0.804) | 0.2107 | 0.732  (0.725-0.740) | 0.704  (0.687-0.721) | 0.740  (0.731-0.748) | 0.421  (0.407-0.435) | 0.903  (0.897-0.909) | 0.527  (0.513-0.541) | 0.530  (0.520-0.541) |
|  | Test | 0.794  (0.777-0.812) | 0.2107 | 0.753  (0.740-0.766) | 0.639  (0.609-0.668) | 0.787  (0.773-0.800) | 0.469  (0.443-0.496) | 0.881  (0.869-0.892) | 0.541  (0.518-0.565) | 0.510  (0.492-0.527) |
| ANN-GB2 | Train | 0.876  (0.870-0.881) | 0.7069 | 0.816  (0.809-0.822) | 0.839  (0.831-0.846) | 0.758  (0.745-0.772) | 0.896  (0.890-0.903) | 0.654  (0.640-0.668) | 0.866  (0.861-0.872) | 0.935  (0.929-0.940) |
|  | Test | 0.893  (0.884-0.902) | 0.7069 | 0.831  (0.820-0.842) | 0.868  (0.857-0.880) | 0.733  (0.708-0.757) | 0.896  (0.885-0.907) | 0.677  (0.652-0.702) | 0.882  (0.873-0.891) | 0.948  (0.939-0.957) |
| ANN-GB2-T | Train | 0.869  (0.864-0.875) | 0.7072 | 0.804  (0.797-0.811) | 0.818  (0.811-0.826) | 0.769  (0.755-0.782) | 0.898  (0.892-0.904) | 0.630  (0.616-0.643) | 0.856  (0.851-0.862) | 0.931  (0.925-0.937) |
|  | Test | 0.889  (0.880-0.898) | 0.7072 | 0.827  (0.815-0.838) | 0.846  (0.834-0.859) | 0.774  (0.750-0.797) | 0.908  (0.898-0.919) | 0.655  (0.630-0.679) | 0.876  (0.868-0.885) | 0.945  (0.936-0.955) |

Table S10

Summary of the performance measures of the models trained using all (24) feature variables, (7) key feature variables, and all except key feature variables. The results shown in the table were calculated by applying models to the testing datasets. -RED: models trained using only key feature variables. -RMV: models trained using all except key feature variables.

| Model | AUROC  (95% CI) | TH | Accuracy  (95% CI) | Sensitivity  (95% CI) | Specificity  (95% CI) | PPV  (95% CI) | NPV  (95% CI) | F1  (95% CI) | AUPRC  (95% CI) |
| --- | --- | --- | --- | --- | --- | --- | --- | --- | --- |
| ANN-GA | 0.942  (0.920-0.965) | 0.0744 | 0.904  (0.895-0.912) | 0.941  (0.902-0.970) | 0.902  (0.893-0.911) | 0.314  (0.277-0.351) | 0.997  (0.995-0.999) | 0.471  (0.428-0.513) | 0.391  (0.368-0.413) |
| ANN-GA-RED | 0.943  (0.921-0.966) | 0.0795 | 0.914  (0.906-0.922) | 0.921  (0.884-0.958) | 0.914  (0.906-0.922) | 0.338  (0.299-0.378) | 0.996  (0.994-0.998) | 0.495  (0.450-0.539) | 0.381  (0.359-0.404) |
| ANN-GA-RMV | 0.697  (0.656-0.738) | 0.0829 | 0.631  (0.616-0.645) | 0.640  (0.574-0.706) | 0.630  (0.616-0.645) | 0.076  (0.064-0.089) | 0.973  (0.967-0.979) | 0.136  (0.114-0.159) | 0.092  (0.051-0.133) |
| ANN-GB1 | 0.824  (0.808-0.841) | 0.2441 | 0.770  (0.758-0.783) | 0.617  (0.587-0.647) | 0.815  (0.802-0.828) | 0.497  (0.469-0.524) | 0.878  (0.867-0.890) | 0.550  (0.525-0.573) | 0.550  (0.534-0.567) |
| ANN-GB1-RED | 0.811  (0.794-0.828) | 0.2039 | 0.753  (0.741-0.766) | 0.741  (0.714-0.768) | 0.757  (0.743-0.771) | 0.474  (0.449-0.498) | 0.908  (0.898-0.919) | 0.578  (0.558-0.600) | 0.510  (0.493-0.527) |
| ANN-GB1-RMV | 0.699  (0.679-0.719) | 0.2088 | 0.696  (0.683-0.710) | 0.583  (0.552-0.613) | 0.730  (0.715-0.744) | 0.389  (0.364-0.413) | 0.855  (0.843-0.868) | 0.467  (0.443-0.492) | 0.445  (0.426-0.465) |
| ANN-GB2 | 0.893  (0.884- 0.902) | 0.7069 | 0.831  (0.820-0.842) | 0.868  (0.857-0.880) | 0.733  (0.708-0.757) | 0.896  (0.885-0.907) | 0.677  (0.652-0.702) | 0.882  (0.873-0.891) | 0.948  (0.939-0.957) |
| ANN-GB2-RED | 0.879  (0.870-0.889) | 0.6975 | 0.815  (0.803-0.826) | 0.842  (0.829-0.854) | 0.744  (0.720-0.769) | 0.897  (0.886-0.908) | 0.639  (0.614-0.664) | 0.868  (0.859-0.877) | 0.938  (0.928-0.948) |
| ANN-GB2-RMV | 0.731  (0.716-0.746) | 0.7324 | 0.666  (0.652-0.679) | 0.672  (0.656-0.688) | 0.649  (0.622-0.676) | 0.836  (0.821-0.850) | 0.427  (0.404-0.449) | 0.745  (0.732-0.758) | 0.868  (0.853-0.883) |
